# AhR and Wnt/β-Catenin Signaling Pathways and Their Interplay

**DOI:** 10.3390/cimb45050248

**Published:** 2023-05-02

**Authors:** Alevtina Y. Grishanova, Lyubov S. Klyushova, Maria L. Perepechaeva

**Affiliations:** Institute of Molecular Biology and Biophysics, Federal Research Center of Fundamental and Translational Medicine, Timakova Str. 2, Novosibirsk 630117, Russia; klyushovals@mail.ru (L.S.K.); perepech@niimbb.ru (M.L.P.)

**Keywords:** AhR, Wnt, β-catenin, crosstalk, mutual interaction

## Abstract

As evolutionarily conserved signaling cascades, AhR and Wnt signaling pathways play a critical role in the control over numerous vital embryonic and somatic processes. AhR performs many endogenous functions by integrating its signaling pathway into organ homeostasis and into the maintenance of crucial cellular functions and biological processes. The Wnt signaling pathway regulates cell proliferation, differentiation, and many other phenomena, and this regulation is important for embryonic development and the dynamic balance of adult tissues. AhR and Wnt are the main signaling pathways participating in the control of cell fate and function. They occupy a central position in a variety of processes linked with development and various pathological conditions. Given the importance of these two signaling cascades, it would be interesting to elucidate the biological implications of their interaction. Functional connections between AhR and Wnt signals take place in cases of crosstalk or interplay, about which quite a lot of information has been accumulated in recent years. This review is focused on recent studies about the mutual interactions of key mediators of AhR and Wnt/β-catenin signaling pathways and on the assessment of the complexity of the crosstalk between the AhR signaling cascade and the canonical Wnt pathway.

## 1. Introduction

### 1.1. AhR

Aryl hydrocarbon receptor (AhR) is a ligand-activated transcription factor [1,2,3]. Initially, AhR was identified in studies on its binding to polychlorinated aromatic hydrocarbons, including 2,3,7,8-tetrachlorodibenzo-*p*-dioxin (TCDD), and polychlorinated biphenyls [1,2,3]. Protection from external chemicals, as implemented by the metabolism of xenobiotics, has long been considered to be AhR’s main function.

AhR is ubiquitously expressed in many vertebrate cell types and mediates various cellular functions. Its numerous physiological and homeostatic functions have been shown by accumulating evidence, including data from experiments on *AhR* knockout mouse models or on orthologs of AhR in nonmammals and experiments with AhR-mediated toxicity of TCDD in mice [4,5,6]. In mouse models, the complete depletion of AhR has been shown to alter the development and function of several organs, including the liver, heart, skin, and immune system [7,8,9,10].

Endogenous functions are modulated by AhR at early stages of embryogenesis during organogenesis in the neuroepithelium, heart, liver, kidneys, lungs, muscles, epidermis, and retina [11]. *AhR* is highly expressed in the tissues of important physiological barriers of the body, such as the placenta, skin, lungs, gastrointestinal tract, and liver [7,12,13].

AhR regulates fundamental metabolic processes that modulate such cellular phenomena as proliferation, differentiation, the cell cycle, adhesion, migration, invasion, pluripotency, and stemness and mediate such physiological functions of AhR as the homeostasis of organs of the cardiac, immune, reproductive, nervous, and intestinal systems and of the liver, insulin–glucose regulation, oxidative stress, and adaptation to hypoxia [14,15,16,17,18,19].

The persistence of AhR in multicellular organisms from mollusks to humans proves that it performs an important physiological function. It is believed that the evolution of AhR, which began hundreds of millions of years ago in multicellular animals, has been driven by adaptation to environmental toxins [20]. Nonetheless, the following hypothesis is likely to be true: the function of AhR was originally the regulation of development, and its involvement in xenobiotic metabolism probably has emerged as an adaptation later, during the evolution of vertebrates [4,20].

### 1.2. Wnt

The signaling pathway of the Wnt (wingless-type MMTV integration site) family of secreted glycolipoproteins is central to multiple developmental processes [21,22]. Compared to the signaling pathways of other growth factors, Wnt signaling has several unique properties and, in fact, governs development and tissue homeostasis. The Wnt signaling pathway takes part in the workings of such crucial cellular phenomena as intercellular communication, cell migration, the determination of the fate of nascent cells, embryogenesis, organogenesis, and homeostasis of embryonic and adult stem cells [21,22,23,24]. The Wnt signaling pathway is evolutionarily conserved and Wnt proteins are active in all clades of the animal kingdom [25].

Between AhR and the Wnt/β-catenin pathways, various types of crosstalk can occur, which have been most fully described in a review by Schneider et al. [26]. In recent years, numerous studies showed potential crosstalk between AhR and Wnt/β-catenin signaling cascades, and a lot of new evidence was accumulated about a dual interaction between these pathways, as did data on the interdependent regulation of their signal transduction as a possible mechanism for the maintenance of physiological or pathophysiological functions. This review covers studies on this topic that have been conducted in recent decades.

## 2. AhR Signaling

### 2.1. The Genomic AhR Signaling Pathway

**Figure 1:**
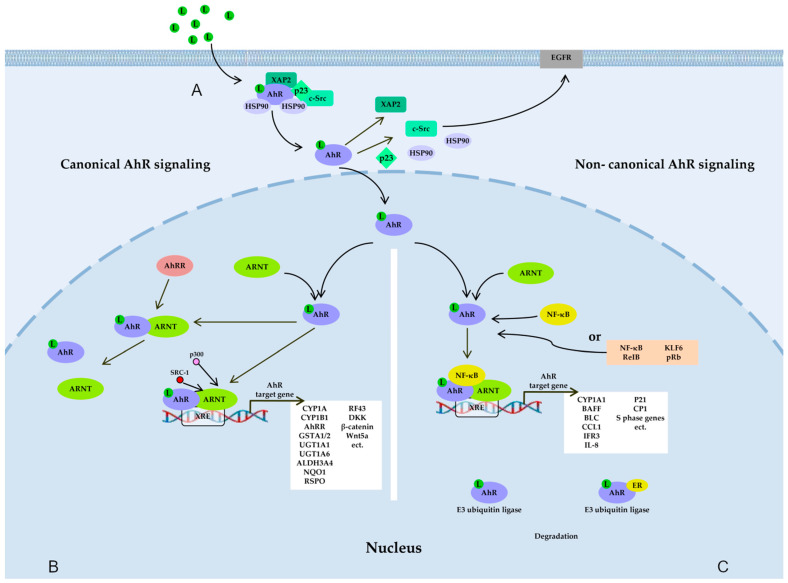
AhR signaling pathways. (**A**) Before ligand binding, AhR is located in the cytosol in the form of a complex with hepatitis B virus X-associated protein 2 (XAP-2), with two heat shock proteins 90 (HSP90), with cochaperone p23, and with additional partners, including c-Src. A ligand changes AhR’s conformation, thereby leading to the dissociation of the complex and translocation of AhR to the nucleus, where AhR forms a dimer either with AhR nuclear transporter (ARNT) (canonical pathway) (**B**) or with partner proteins other than ARNT (noncanonical pathway) (**C**), such as transcription factor Krüppel-like factor 6 (KLF6), transcription factors of the nuclear factor kappa B (NF-κB) family, retinoblastoma protein (pRb), or nuclear receptors (e.g., estrogen receptor α). The resultant dimer binds to a xenobiotic-responsive element (XRE) in DNA and, thus, induces the transcription of target genes of AhR. AhR also takes part in nongenomic signaling: when dissociated from the c-Src complex, AhR can interact with epidermal growth factor receptor (EGFR), whose downstream signaling includes the focal adhesion kinase FAK pathway and mitogen-activated protein kinase (MAPK) pathways called RAS–RAF–MEK1/2–ERK1/2 and AKT–PI3K–mTOR, as well as protein kinase C (PKC), STAT proteins, SRC, and NF-κB. AhR is also a component of a protein complex that functions as an E3 ubiquitin ligase.

The first identified function of AhR (related to its participation in the metabolism of xenobiotics) is implemented through a genomic pathway. AhR is a member of the basic helix-loop-helix/Per-Arnt-Sim (bHLH-PAS) transcription factor superfamily. In its inactive basal state, AhR is a part of a cytoplasmic complex composed of two chaperones—heat shock protein 90 (Hsp90) and X-associated protein 2 (XAP2) [also known as AhR-interacting protein (AIP)/ARA9]—as well as cochaperone p23 and additional partners, including kinases c-Src [5,27,28,29] (Figure 1A).

When a ligand binds to AhR, this event induces the dissociation of the complex, thereby leading to the potential activation of several AhR pathways.

#### 2.1.1. The Canonical AhR Signaling Pathway

The canonical pathway (Figure 1B), which is genomic, starts after the translocation of AhR into the nucleus and dimerization with its partner, ARNT. The AhR/ARNT heterodimer can bind to specific response elements called xenobiotic response elements (XREs), located in the regulatory sequences of various target genes. XREs have the consensus sequence 5′-TNGCGTG-3′, in which AhR recognizes the half site (TNGC), whereas ARNT recognizes GTG [28,30,31,32]. After binding to a ligand, the complex dissociates, resulting in the potential activation of several pathways: the genomic pathway involving its nuclear partner ARNT and the regulation of target gene transcription [28,32] and nongenomic signaling [33,34].

Among the target genes containing XRE in their promoters are genes coding for xenobiotic biotransformation enzymes CYP1A1, CYP1A2, CYP1B1, UGT1A1, UGT1A6, GSTA1/2, and ABCG2, which mediate the detoxification function of AhR [1,2,3], as well as genes encoding murine epiregulin, ecto-ATP, δ-aminolevulinic acid synthase, prostaglandin endoperoxide H synthase 2, MDR1, BRCP, AhRR, and p27kip1 [32] and EGFR ligands called amphiregulin (AREG) and epiregulin (EREG) [4].

#### 2.1.2. “Noncanonical” AhR Signaling Pathways

Other “noncanonical” pathways (Figure 1C) after the dissociation of the cytoplasmic complex potentially lead to the interaction of AhR with other partners such as transcription factor Krüppel-like factor 6 (KLF6) or transcription factors of the nuclear factor kappa B (NF-κB) family (e.g., RelB) [35,36], retinoblastoma protein (pRb), or nuclear receptors (e.g., estrogen receptor α) [37,38,39,40]. Proteins AhR and KLF6 form a heterodimer that recognizes a novel nonconsensus XRE (NC-XRE) and initiates the transcription of the genes involved in cell cycle regulation. Proteins AhR and RelB (an NF-κB subunit) combine into a heterodimer that recognizes a RelB–XRE complex and induces the transcription of some chemokine genes [41,42,43]. AhR and NF-κB form a heterodimer that leads to the induction of the expression of cytokines and chemokines: B-cell-activating factor of the tumor necrosis factor family (BAFF), B-lymphocyte chemoattractant (BLC), CC-chemokine ligand 1 (CCL1), and interferon-responsive factor (IFR3) [44,45]. The AhR–ARNT–NF-κB interaction decreases the expression of *CYP1A1*. AhR and pRb form a heterodimer that results in the blockade of cell cycle progression by downregulating S-phase genes [40,46,47,48].

Among the genes that are targets of the noncanonical pathway, there are those encoding proteins responsible for organ development (p21^Cip1^, p27^KIP1^, p40^phox^, and Blimp1), for antimicrobial defense (NADPH oxidase/p40^phox^), for immunity/inflammation (C-Kit, Il-1β, Il-6, IL-17A, Il-22, CXCL5, and IDO/TDO), for energy homeostasis (TiPARP, CD36, and CD38), and for reproduction (CYP19) [48].

### 2.2. Nongenomic AhR Signaling

In addition to the genomic signaling through target genes [49,50,51,52,53], AhR participates in nongenomic signaling [33,34]. For instance, a member of the c-Src cytoplasmic complex, after activation of AhR and dissociation from the complex, can trigger alternative pathways by activating signaling via EGFR, which is a receptor tyrosine kinase (RTK) of the ErbB family [54] and plays a key part in embryonic development and physiology [55] and in several cellular processes such as proliferation, differentiation, and apoptosis. The signaling downstream of EGFR includes the focal adhesion kinase (FAK) pathway and pathways of mitogen-activated protein kinase (MAPK) RAS-RAF-MEK1/2-ERK1/2 and AKT-PI3K-mTOR, as well as pathways of protein kinase C (PKC), STAT, SRC, and NF-κB; these may participate in the modulation of proliferation and adhesion processes and of inflammation [4,34,56,57]. Aside from acting as a ligand-activated transcription factor in gene expression, AhR is also a component of a protein complex that functions as an E3 ubiquitin ligase for the proteasomal degradation of substrate proteins [58,59].

### 2.3. AhR Ligands

AhR regulates many functions owing to the huge variety of ligands that activate it. The activity of AhR depends on the type of ligands, which are xenobiotics, naturally occurring food compounds, and endogenous molecules [17,60,61,62].

Numerous ligands of AhR have been identified as agonists of AhR, including synthetic and environmental chemicals [60,61]. Nonetheless, the subsequent discovery of endogenous and plant source ligands led to the recognition that AhR plays a fundamental role in normal cell biology and physiology by ensuring normal development and homeostasis [63,64,65,66,67,68,69,70].

#### 2.3.1. Exogenous Synthetic AhR Ligands

Xenobiotics, which are mainly aromatic hydrocarbons, were the first discovered ligands of AhR. The best known exogenous synthetic ligands are halogenated aromatic hydrocarbons, including TCDD, and polycyclic aromatic hydrocarbons, such as 3-methylcholanthrene (3MC) and benzo(a)pyrene (BaP), which have strong affinity for AhR [1,2,61,69]. These compounds are present in the air as complex mixtures and are very stable; some of them can accumulate in the body. Prolonged exposure leads to pronounced toxic effects, including immunotoxicity, thyroid dysfunction, anomalies of nervous-system development, and impairment of reproductive functions [11,28,61].

#### 2.3.2. Natural AhR Ligands

The natural compounds that are natural AhR ligands present in food can activate AhR and have been characterized as ligands of AhR, although most of them are low-affinity ligands [63,71,72]. This is the most widespread class of polyphenols—flavonoids quercetin and resveratrol found in fruits and vegetables—and indoles such as indole-3-carbinol (I3C) or one of its secondary metabolites, 3,3′-diindolylmethane, obtained from cruciferous vegetables [28]. Quercetin and resveratrol activate AhR [73,74] and have both agonistic and antagonistic effects [11,28,75].

#### 2.3.3. Endogenous AhR Ligands

Finally, the endogenous molecules that are formed by endogenous metabolism can also serve as ligands of AhR. Endogenous ligands that are capable of activating AhR include indole and its derivatives such as tryptophan and its derivatives tryptamine and kynurenine (Kyn) pathway metabolites [indole-3-acetic acid (IAA), Kyn, and FICZ (formylindolo[3,2-b]carbazole)], as well as leukotrienes and arachidonic-acid metabolites [lipoxin A4, some prostaglandins (PGE2), and heme breakdown products: bilirubin and biliverdin] [11,76,77,78]. It is worth mentioning that the tryptophan photoproduct FICZ, which arises in the skin under ultraviolet irradiation, is the most potent AhR ligand known to date. It has a stronger affinity for human AhR than TCDD does [11].

Some of the tryptophan metabolites originate from the microbiota and are agonists or antagonists of AhR activity (indole, IAA, or tryptamine) [11,68,75,79,80]. Indirubin and indigo represent another family of phenols that are present in our body under normal physiological conditions and are strong inducers of AhR [77,81]. Some compounds of bacterial origin are also known to be ligands of AhR. These are virulence factors (phenazine or pyocyanin from *Pseudomonas aeruginosa*) [80] or modulators (lipopolysaccharide from *Escherichia coli*) [82].

Thus, AhR acts as a receptor for a variety of exogenous and endogenous molecules and can mediate either toxic or physiological effects. Apparently, the nature of a ligand, a tissue- and organ-specific microenvironment, and the presence of coregulators or specific transcription factors in the cell contribute to the outcome of the modulation of gene transcription by AhR. The diversity of responses resulting from the activation of AhR may be explained by various interactions of AhR with other proteins or transcription factors [83].

So far, numerous proteins have been described that affect the activity of AhR and vice versa [28]. AhR, by performing functions in the regulation of transcription, participates in various key signaling pathways that eventually affect the homeostasis of organs and tissues in response to either endogenous or exogenous stimuli. For instance, an AhR pathway can directly functionally interact with the signaling pathways involved in organ development, e.g., the TGFβ/BMP (transforming growth factor β/bone morphogenetic protein) pathway and the pathways of receptor tyrosine kinases—including KGFR (keratinocyte growth factor receptor), VEGFR (vascular endothelial growth factor receptor), and EGFR (epidermal growth factor receptor)—as well as the Notch pathway and Wnt/β-catenin signaling [14]. The canonical Wnt/β-catenin pathway is examined in detail in this review.

## 3. Wnt Signaling

The Wnt signaling pathway constitutes a highly complex regulatory network. Three intracellular Wnt signaling pathways have been identified, which include the crucial and best-studied canonical pathway—i.e., the Wnt/β-catenin pathway, which activates gene transcription via β-catenin—and noncanonical pathways [21,22]. Canonical Wnt signaling functions via the regulation of the amount of a transcription coactivator known as β-catenin (CTNNB1), which controls the expression programs of key genes associated with ontogenesis. Noncanonical Wnt/PCP pathways (planar cell polarity pathways) and Wnt/calcium (Wnt/Ca^2+^) pathways regulate cytoskeletal dynamics and Ca^2+^-dependent pathways (Wnt/Ca^2+^ pathway) that influence cell adhesion and the expression of related genes via an intracellular Ca^2+^ release [21,22,23,24]. The Wnt signaling pathway is activated by a ligand protein binding to plasma membrane receptors called Frizzled and coreceptors [84].

Canonical ligands called Wnt mediate the post-translational regulation of β-catenin and induce the accumulation of β-catenin, which, after translocation to the nucleus, triggers the expression of Wnt-responsive target genes [21,23]. β-catenin is a multifunctional protein present in various subcellular components. The membrane-bound form of β-catenin interacts with E-cadherin and fuses actin filaments, thereby giving rise to the cytoskeleton. The cytosolic form of β-catenin comes into being after a release of the bound form into the cytosol by tyrosine phosphorylation and acts as a downstream protein of the Wnt signaling pathway [85].

**Figure 2:**
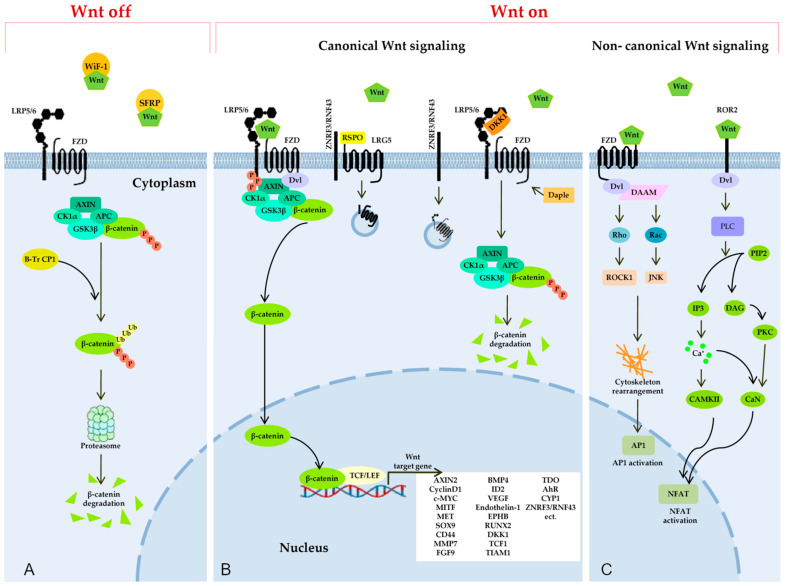
Wnt signaling pathways. (**A**) Secreted Frizzled-related proteins (SFRPs) and Wnt-inhibitory factor 1 (WIF1) counteract Wnt signaling by isolating the Wnt protein in the extracellular space. In the absence of Wnt proteins, degradation of β-catenin takes place in the cytoplasm, and this process limits the transcription of Wnt target genes. The degradation of cytosolic β-catenin is mediated by proteasomes or autophagy because of persistent phosphorylation of β-catenin by a multimeric protein degradation complex containing Axin, adenomatous polyposis colonic (APC) scaffold proteins, casein kinase 1α (CK1α), and glycogen synthase kinase 3 (GSK3) and because of subsequent ubiquitination by β-transducin repeat-containing protein 1 (β-TrCP1). (**B**) Binding of a Wnt protein (usually Wnt3A and Wnt1) to a receptor called Frizzled (FZD) and to coreceptor LRP5/6 induces phosphorylation of the cytoplasmic region of LRP5/6 by GSK3β and recruitment of a cytosolic protein called Disheveled (Dvl). This event leads to an increase in the amount of β-catenin in the cytoplasm, owing to isolation and inhibition of the protein complex responsible for β-catenin phosphorylation. After translocation to the nucleus, β-catenin forms an active complex with transcription factor T-cell factor and a transcription factor from the lymphoid enhancer-binding factor family (TCF/LEF) and other histone-modifying coactivators (CBP, p300, and BCL9) and initiates the transcription of target genes of Wnt. The Daple protein may inhibit the recruitment of DVL. A secreted inhibitor by the name of Dickkopf (DKK) can also counteract Wnt signal transduction by competitively binding to LRP5/6. Ubiquitin ligases ZNRF3/RNF43 reduce the availability of membrane receptors (LRP5/6) and FZD to Wnt through their internalization and degradation, thereby negatively regulating the Wnt pathway. R-spondin (RSPO) binds to receptor proteins called leucine rich repeat-containing G protein-coupled receptor 4, 5, or 6 (LGR4/5/6) and to the extracellular domain of RNF43/ZNRF3, resulting in their physical association, internalization into the cytoplasm, and the removal of ZNRF3/RNF43 from the membrane. In this way, the availability of the Wnt membrane receptor increases, and the activation of the Wnt ligand–mediated pathway is enhanced. (**C**) Binding of Wnt isoforms (e.g., Wnt-5a) to either FZD or Ror2 triggers noncanonical (β-catenin–independent) Wnt signaling cascades, including the inhibition of the canonical Wnt/β-catenin pathway. In the planar cell polarity (PCP) pathway, Wnt binds to receptors FZD, thereby activating Dvl and Dvl-associated activator of morphogenesis (DAAM). Dvl and DAAM together trigger small GTPases Rho and Rac, which catalyze the activation of JNK and ROCK1, resulting in a cytoskeleton rearrangement and changes in gene expression via activation of the activating protein-1 (AP1) family of transcription factors. In the Wnt/Ca^2+^ pathway, a signal is transmitted via a triggering of phospholipase C (PLC) through Dvl activation. PLC hydrolyzes phosphatidylinositol 4,5-bisphosphate (PIP2) generating inositol 1,4,5-triphosphate (IP3) and 1,2-diacylglycerol (DAG). IP3 activates calcium/calmodulin-dependent protein kinase II (CAMKII) and calcineurin (CaN) through a release of calcium from the endoplasmic reticulum. Active CAMKII and CaN alter a target gene’s expression by inducing a transcription factor called nuclear factor of activated T cells (NFAT). DAG in turn triggers protein kinase C, which raises CaN activity.

In the absence of ligand proteins (Figure 2A) (Wnt), cytosolic β-catenin is cleaved by a destruction complex consisting of scaffold proteins [adenomatous polyposis of the colon (APC), casein kinase 1α (CK1), and glycogen synthesis kinase 3 (GSK3)] situated on an Axin protein “platform” Axin and thus maintaining low cytoplasmic levels of β-catenin in the cell [86]. In this multiprotein complex, β-catenin is phosphorylated by GSK3β and cleaved via the ubiquitin-proteasome system. GSK3β is one of the most complicated kinases for cellular processes, and its possible substrates are hundreds of proteins, one of which is β-catenin [87]. GSK3β is recognized as a key player in the modulation of the β-catenin function [86,87]. The destruction complex induces the continuous elimination of β-catenin, thereby preventing its nuclear transport, so that the expression of Wnt-responsive genes is switched off.

### 3.1. The Canonical Wnt Signaling Pathway

In the canonical Wnt signaling pathway (Figure 2B), ligands called Wnt bind to several different receptors, thus promoting a variety of cellular processes depending on the presence of coreceptors and on the assembly of receptor complexes [88]. The Wnt/β-catenin signaling pathway is regulated at the cell surface by multiple secreted proteins, including two major families of endogenous agonists of the Wnt signaling pathway: proteins Wnt and R-spondin (RSPO) [89,90].

The Wnt/β-catenin signaling pathway begins with the formation of a complex of Wnt with a transmembrane cell surface receptor called Frizzled (FZD) and coreceptors: membrane complexes LRP5/6 related to low-density lipoprotein receptor. This receptor complex recruits the destruction complex to the cell membrane via an interaction of Axin with a phosphorylated LRP5/6 tail and with multifunctional phosphoprotein Disheveled (Dvl), which facilitates the interaction between LRP5/6 and Axin and mediates the inhibition of the β-catenin destruction complex [89,91].

In this way, it prevents constitutive proteasome-dependent degradation of β-catenin either by inhibiting the phosphorylation of β-catenin in combination with the disassembly of the destruction complex or through the inactivation of ubiquitination and proteasomal degradation of β-catenin in the destruction complex. As a consequence, free β-catenin accumulating in the cytoplasm enters the nucleus and initiates the transcription of Wnt-sensitive target genes with the help of transcriptional T-cell factors and factors of the lymphoid enhancer-binding factor family (TCF/LEF) [92,93,94,95]

RSPO, another endogenous agonist of Wnt signaling, is a secreted glycoprotein that interacts with low-density lipoprotein receptor proteins 4/5/6 (LGR4/5/6), thereby substantially enhancing Wnt/β-catenin signaling [89,90,96].

The negative regulation of Wnt signaling is mediated by other secreted factors, including secreted Frizzled-related proteins (sFRPs), Wnt-inhibitory factor 1 (WIF-1), the Dickkopf family (DKK), and ubiquitin ligases ZNRF3/RNF43 [97]. sFRP and WIF-1 directly serve as antagonists of Wnt proteins by binding them in the extracellular space [98].

DKK can associate with LRP5/6 proteins to prevent the formation of the FZD–Wnt–LRP complex. The DKK protein (belonging to a protein family containing four members), when binding to LRP5/6, stabilizes cytosolic β-catenin by suppressing the Axin function. The complex of DKK with LRP5/6 inhibits Wnt signaling in a competitive way toward Wnt proteins or in a way where DKK separates LRP5/6 from the plasma membrane. Thus, DKK regulates the LRP5/6 amounts, thereby coordinating various biological processes depending on the Wnt signal [97,99].

Wnt/β-catenin signaling negative regulators called ubiquitin ligases ZNRF3/RNF43 inhibit i) the binding of RSPO to LGR5 and ii) stimulation of the canonical Wnt pathway, thus reducing the availability of Wnt receptors on the membrane via internalization and the degradation of membrane receptors FZD and LRP5/6 [100,101,102,103,104]. The LGR5–RSPO–RNF43/ZNRF3 regulatory axis further complicates the Wnt/β-catenin pathway.

### 3.2. The Noncanonical Wnt Signaling Pathway

Binding of Wnt isoforms to either FZD or Ror2 triggers noncanonical, i.e., β-catenin–independent Wnt signaling cascades (Figure 2C), including the inhibition of the canonical Wnt/β-catenin pathway. In the planar cell polarity (PCP) pathway, Wnt binds to receptors FZD, thereby activating Dvl and Dvl-associated activator of morphogenesis (DAAM). Dvl and DAAM together trigger small GTPases Rho and Rac, which catalyze the activation of JNK and ROCK1, resulting in a cytoskeleton rearrangement and changes in the gene expression.

In the Wnt/Ca^2+^ pathway, a signal is transmitted via a triggering of phospholipase C (PLC) through Dvl activation. PLC hydrolyzes phosphatidylinositol 4,5-bisphosphate generating inositol 1,4,5-triphosphate and 1,2-diacylglycerol. Inositol 1,4,5-triphosphate activates calcium/calmodulin-dependent protein kinase II (CAMKII) and calcineurin (CaN) through a release of calcium from the endoplasmic reticulum. Active CAMKII and CaN alter a target gene’s expression by inducing a transcription factor called nuclear factor of activated T cells (NFAT). DAG triggers the protein kinase C, which raises the CaN activity.

### 3.3. Wnt Target Genes

A growing number of endogenous target genes of Wnt have been identified [105], in particular β-catenin–responsive target genes. These targets include the genes required for cell proliferation, differentiation, adhesion, survival, migration, self-renewal, metabolism, and epithelial–mesenchymal transition (EMT) [106], as well as genes of *Axin2* and *RNF43/ZNRF3*, which are components of the Wnt/β-catenin pathway that constitute negative feedback loops in its regulation [104].

## 4. Intersection of AhR and Wnt Signaling

### 4.1. Effects of AhR on Wnt Signaling

#### 4.1.1. The Influence of AhR (Not Activated by Agonists) on Wnt Signaling

A review by Schneider et al. [26] fully described the then-known evidence of mutual interference between two pathways: AhR and β-catenin cascades [26]. AhR and Wnt/β-catenin have been shown to cooperate in the induction of transcriptional targets of AhR, and, in this context, the persistent activation of AhR causes a decrease in the level of active β-catenin, thereby influencing the phenotype of liver progenitor cells and leading them to more differentiated cell types [107]. AhR has been shown to be an inhibitor of canonical Wnt signaling in the mouse gut and to be capable of suppressing gut carcinogenesis through the degradation of β-catenin [108]. In a human breast cancer cell line, the constitutive expression of AhR via the introduction of a mutation can downregulate CTNNB1 [26].

Further research has revealed that the loss of AhR signaling in murine intestinal lamina propria CD11c^+^ antigen-presenting cells is related to aberrant Wnt signaling in these cells and to the abnormal development of the intestinal epithelium [109]. In that study, overall upregulation of Wnt target genes such as *Axin2*, *Lgr5*, *c-Myc*, and *Nmp4* was noted. The expression of Dkk3 is significantly elevated in AhR-deficient intestinal macrophages [109]. Furthermore, elevated levels of β-catenin in the AhR-null (*AhR*^−/−^) liver have been found in preweaning-to-adult mice, and it has been demonstrated that by inhibiting Wnt/β-catenin signaling along with PI3K and ERK signaling, AhR contributes to the physiological polyploidization of the liver [110]. AhR is required for the proper maturation, differentiation, and polyploidization that take place during the transition from an immature to mature liver, thus ultimately leading to an organ with nonproliferative cells. Accordingly, the *AhR*^−/−^ liver phenotype includes a faster proliferation with deficient polyploidization and the activation of signaling cascades, including Wnt/β-catenin, PI3K/AKT, and ERK pathways. This change is probably possible because AhR is a component of a repressive complex that binds to β-catenin, thereby promoting its ubiquitination and subsequent degradation [108].

Because both proteins are coimmunoprecipitated under normal conditions in the adult liver [110], AhR may contribute to the regulation of liver repair and to the blockage of carcinogenesis via β-catenin signaling and the modulation of stem cells [111]. A study [111] indicates that AhR deficiency improves liver regeneration after acute toxic injury by CCl_4_ but promotes the development of hepatocarcinoma, probably because of the expansion of stem cells and the activation of factors associated with pluripotency, including β-catenin. The involvement of β-catenin signaling in AhR-dependent liver regeneration is confirmed by such observations as increased levels of β-catenin after the CCl_4_ injury in *AhR*^−/−^ mice but not in *AhR*^+/+^ mice, the activation of Axin2 and repression of *Dkk1* in the *AhR*^−/−^ liver, and *Dkk1* induction in the *AhR*^−/−^ liver when regeneration reaches its maximum [111].

Thus, AhR and β-catenin are probably linked within a regulatory network that controls the formation of stem-like and pluripotent cells necessary for liver regeneration. This supposition is supported by a finding that the activation of AhR alters Wnt/β-catenin signaling, thereby impairing tissue regeneration in zebrafish and impeding urogenital-sinus formation during prostate development [26,112,113].

AhR also takes part in liver regeneration in adult rodents. The application of 2/3 partial hepatectomy to *AhR*^+/+^ and *AhR*^−/−^ livers has revealed that the AhR depletion improves liver regeneration in response to severe damage to this organ, and this benefit is most likely mediated by the expansion of hepatic stem cells, which are already known to improve regeneration after acute toxic injury. Wnt/β-catenin, PI3K/AKT, and Hippo signaling pathways appear to participate in the regulatory process in an AhR-dependent manner [111,114].

AhR plays a critical role in intestinal stem cells (ISCs) by calibrating their response to Wnt-β-catenin signals, thereby enabling coordinated stem cell renewal and differentiation [115]. Using *AhR*^−/−^ mouse models and intestinal organoid cultures, those authors found that AhR acts directly on ISCs by regulating the expression of E3 ubiquitin ligases *RNF43* and *ZNRF3* (which suppress Wnt/β-catenin signaling), thus limiting the excessive proliferation of ISCs. In mice having intact AhR, these defects can be reversed via the activation of AhR by means of nutritional ligands, which was found to restore the regulation of the Wnt/β-catenin pathway and barrier homeostasis and to prevent tumorigenesis through the transcriptional regulation of ubiquitin ligases *RNF43* and *ZNRF3* [115].

AhR signal transduction suppresses Wnt/β-catenin signaling in colon cells, thereby playing a protective role in genetically induced colon carcinogenesis [116]. The enhancement of Wnt signaling by an *AhR* knockout in the murine intestinal epithelium promotes the expansion and clonogenic capacity of colonic stem/progenitor cells carrying mutations Apc^S580/+^ and Kras^G12D/+^.

Shackleford G. et al. have reported that AhR can interact with β-catenin and apparently may stimulate its degradation during the peripheral myelination of nerves [117]. This conclusion is based on evidence that an *AhR* knockdown in mice alters the β-catenin expression in the sciatic nerve, whereas an *AhR* knockdown in the MSC80 mouse Schwann cell line activates the Wnt/β-catenin pathway. For instance, the protein level of active β-catenin and mRNA expression of components of the Wnt signaling pathway (*Lrp6*, *Dvl2*, *Dvl3*, and *Axin2*) increase, relevant sites in the TCF/LEF promoter are activated, and a protein–protein interaction of β-catenin with AhR is detectable by immunoprecipitation [117].

The AhR signaling pathway elevates the expression of β-catenin and of *Wnt5a/b* in the tumor tissue of patients with inflammatory breast cancer (IBC) that overexpresses AhR and its target gene/protein *CYP1B1* [118]. In that study, it was shown that a knockdown of the *AhR* gene causes the suppression of *CYP1B1* and *Wnt5a* expression in the IBC cells.

#### 4.1.2. Effects of Activated AhR on Wnt Signaling

##### Upregulation of Wnt Signaling

In early studies by L.K. Mathew and coworkers [113] concerning the effects of AhR agonist TCDD on Wnt signaling in zebrafish, they showed that the activation of AhR by TCDD enhances canonical Wnt signaling through the overexpression of R-Spondin1 (Rspo1) [113]. This Rspo1 signaling is mediated by a coreceptor called LRP6, which launches the Wnt pathway and leads to the inhibition of regeneration in zebrafish under the influence of TCDD.

The administration of TCDD to the embryonic chick thymus causes a change in the Wnt signaling pathway owing to the initiation of Wnt protein expression via an AhR-dependent pathway. The activation of genes encoding proteins WNT5A, WNT2B, and WNT7B has been registered [119].

In human poorly differentiated colon carcinoma cell line RKO in vitro, TCDD-activated AhR causes an increase in the β-catenin levels. TCDD suppresses the growth of RKO cells and promotes their death through AhR signaling, likely as a result of the stimulation of multiple molecules involved in the modulation of various signaling pathways, including via β-catenin [120]. When these data are compared with findings from another study on human colon cancer cell lines DLD-1, SW480, and HCT116, there is a controversy regarding whether AhR agonists enhance or weaken β-catenin and its signal [121]. In these cell lines, there was no effect of such AhR ligands as 3MC, I3C, IAA, and β-naphthoflavone (βNF) on the amount of β-catenin. On the contrary, only a unidirectional interaction was found, which is the upregulation of the AhR signal by β-catenin [121].

In human liver cancer HepG2 cells, TCDD-activated AhR in vitro induces an increase in β-catenin levels along with STAT3, Ras, and Akt amounts; these proteins participate in cell proliferation and differentiation [122].

Many tumors contain a small population of cells that have a high potential for tumor initiation, stem cell–like properties, and capacity for self-renewal and chemoresistance. A research article about cancer stem cells (CSCs) from human choriocarcinoma cell line JEG-3 revealed that the activation of AhR by TCDD induces the expression of β-catenin and its nuclear translocation and initiates the expression of β-catenin targets: cyclin D1 and c-Myc [123]. The launch of the β-catenin pathway implies the involvement of a Wnt signaling cascade in the effects of AhR on choriocarcinoma CSCs.

Al-Dhfyan and colleagues [124] demonstrated that the activation of AhR/CYP1A1 in breast cancer MCF-7 cells by TCDD or 7,12-dimethylbenz[a]anthracene induces the overexpression of β-catenin and its downstream target cyclin D1 at mRNA and protein levels. The activation of the β-catenin pathway and Akt is mediated by the AhR/CYP1A1 signaling cascade and has been shown to mediate the proliferation and chemoresistance of mammary gland CSCs [124]. The activation of AhR by TCDD in wild-type human ovarian cancer A2780 cells and cisplatin-resistant cells (A2780cis) indicates that AhR mediates the properties, self-renewal, and maintenance of CSCs and metastatic potential by launching PI3K/Akt and Wnt/β-catenin signaling pathways and mediates chemoresistance by preventing apoptosis [125]. An earlier paper about breast cancer MCF-7 cells showed that the activation of AhR/CYP1A1 promotes chemoresistance through the triggering of Wnt/β-catenin and ALDH pathways, which mediate the development, maintenance, and self-renewal of CSCs [124].

A report [125] revealed that the activation of AhR induces Wnt/β-catenin pathways in wild-type A2780 cells but not in chemoresistant cells (A2780cis), indicating that other pathways may be involved. It has been demonstrated that, in breast cancer cell lines MCF-7 and MDA-MB-231 under the influence of organophosphate pesticide chlorpyrifos (CPF) and due to the action of AhR, cell proliferation is induced and the Wnt/β-catenin signaling pathway is launched, partially via the PGE2 pathway [126]. It was shown there that CPF, by the activation of AhR, enhances the expression of β-catenin and diminishes GSK3β activity [126].

It has been found that AhR activated by its endogenous ligand FICZ launches Wnt/β-catenin signaling in periodontal ligament cells in humans and mice, thus causing mineralization in the tissue in question [127]. In these cells, an AhR antagonist, StemRegenin 1 (SR1), causes the opposite outcome [127]. A study of the impact of FICZ on neurogenesis and on its interaction with the Wnt/β-catenin signaling pathway in mouse hippocampal tissue suggests that treatment with FICZ leads to the β-catenin upregulation at mRNA and protein levels [128]. AhR and WNT/β-catenin have also been shown to play a modulatory role in beta-amyloid precursor protein (APP) expression and affect hippocampus-dependent learning and memory deficits [129].

AhR–Wnt/β-catenin crosstalk in fetal heart tissues has been demonstrated by combined treatment with two AhR activators: cadmium (Cd) and FICZ [130]. In that study, mouse fetuses were exposed either to Cd alone, which can activate both AhR and Wnt/β-catenin signaling pathways, or to a combination of Cd with FICZ on gestational day zero. In fetal heart tissues, the mRNA expression levels of the target genes of Wnt/β-catenin went up in the presence of FICZ. During combined exposure to Cd and FICZ, AhR proved to be overexpressed in fetal hearts and the mRNA expression levels of Wnt/β-catenin target genes—*Ctnnb1* and *Nkx2.5* (but not *Gsk3β*)—also rose significantly. In this context, it was shown that the dysregulation of AhR–Wnt/β-catenin signaling during cardiogenesis can affect the development of the mouse heart. These data indicate that chemical contaminants such as Cd, by inhibiting the metabolic degradation of FICZ, may interfere with the normal function of AhR, which plays a physiological role in the regulation of Wnt/β-catenin.

It is reported that AhR can promote the EMT mediated by β-catenin, and this EMT contributes to the onset and progression of pulmonary fibrosis [131,132]. In a study by P.C.M. Selvam et al. [133], it was found that the activation of AhR by an AhR agonist such as TCDD, indeno(1,2,3-cd)pyrene, or FICZ promotes the production of inflammatory cytokines in the mouse macrophage cell line RAW 264.7 and the induction of the expression of biomarkers of EMT in the mouse lung epithelial cell line MLE-12. They demonstrated that such production of inflammatory factors was achieved through an influence on the Wnt/β-catenin signaling pathway, and indeno(1,2,3-cd)pyrene turned out to be a more effective agonist, which led to the accumulation of active β-catenin in the cytoplasm, thereby creating a microenvironment that induces EMT. Overall, their results indicated that the EMT was mediated by the activation of AhR after exposure to AhR agonists through a launch of the Wnt/β-catenin pathway. Nonetheless, the mechanisms or pathways taking part in the activation of this Wnt/β-catenin signaling have not yet been identified [133].

In an in vitro model of intestinal organoids derived from intestinal crypts of C57BL/6 mice, investigators studied the effects of an anticancer phytochemical ligand of AhR (I3C) on intestinal organoid development [134]. It was noted that I3C raised the level of active nonphosphorylated β-catenin but suppressed a Notch signal. This report provides direct evidence for the role of AhR in the regulation of intestinal stem cell development. For instance, I3C elevated the expression of Muc2 and lysozyme (i.e., clone-specific genes of goblet cells and Paneth cells, respectively) but suppressed the expression of IAP: a marker gene of enterocytes. In the intestines of mice treated with I3C, the number of goblet cells decreased, but the number of Paneth cells and the depth and length of crypts and villi did not change [134]. Those authors have previously reported that the activation of AhR by TCDD or FICZ, on the contrary, inhibits Wnt signaling by reducing β-catenin protein levels in intestinal epithelial organoids of C57BL/6 mice and suppresses intestinal organoid development [135].

In a paper by B. Liu et al. [136] on the mechanism of melanin synthesis in cultured normal human epidermal melanocytes under the action of an AhR ligand called astragaloside IV (AS-IV, which is a natural flavonoid), it was shown that AS-IV stimulates melanin synthesis. This induction of melanogenesis by AS-IV was at least partially mediated by AhR-dependent AKT–GSK3β–β-catenin signaling. Elevated phosphorylation of AKT and of GSK3β was identified in the normal human epidermal melanocytes treated with AS-IV. An increased concentration of β-catenin in the nucleus was observed, as was the significantly enhanced phosphorylation of AKT and of GSK3β. These findings are consistent with the notion of prevention of β-catenin degradation, an increase in β-catenin stability in the cytoplasm, and β-catenin translocation to the nucleus as a consequence of phosphorylation of components of the inhibitory complex: AKT and GSK3β [136].

High-throughput RNA sequencing in colon cancer cell lines has identified a signature of AhR target genes regulated by exogenous ligands of AhR of the TCDD type and by endogenous ligand Kyn, a tryptophan metabolite. Among these genes, along with *CYP1A1*, *ALDH1A3*, *ABCG2*, and *ADGFR1*, the gene of actin-uncoupling protein scinderin (*SCIN*) was identified [137]. SCIN, which is a Ca^2+^-dependent actin filament–uncoupling protein, proved to be required for cell proliferation. The suppression of SCIN limited cell proliferation, while its expression increased it. The researchers revealed a unique link between SCIN (whose expression is regulated by TCDD or by the endogenous AhR agonist Kyn) and WNT signaling. It was noted that the amount of β-catenin in cells went up with the increased expression of SCIN, and that SCIN expression is high in a variety of samples from patients with colon cancer, which also contained elevated levels of β-catenin. Notably, SCIN expression promotes the nuclear translocation of β-catenin and thereby triggers the WNT pathway. Those authors described a novel signaling mechanism in which SCIN, probably owing to its ability to modify the actin cytoskeleton, facilitates the nuclear translocation of β-catenin [137].

In cellular models of hepatocellular carcinoma (HCC) with an inducible knockdown (in HuH-7 and Sk-Hep1 cells), it has been shown that the enhancement of AhR and β-catenin pathways is coordinated and is a consequence of the activation of an enzyme called **IDO1**, which catalyzes the formation of the tryptophan metabolite Kyn. After a triggering of IDO1 and the subsequent activation of AhR, β-catenin is activated [138]. The results of that study uncovered the previously unknown involvement of IDO1 in HCC and pointed to a new mechanism by which IDO1 has an oncogenic influence on HCC cells via two pathways. The first one is a proliferative cascade that is primarily triggered by AhR–Src–PTEN–PI3K/Akt–GSK3β–β-catenin signaling, and the other one is a prometastatic pathway that relies on the cooperation of AhR/β-catenin and Snail [138].

Nevertheless, in mouse and human tumor intestinal epithelia, Kyn has been shown to promote β-catenin activity via a PI3K–AKT–GSK3β signaling event, not by AhR-mediated transcriptional activity [139]. Recent papers revealed the overexpression of one of the key enzymes of the Kyn pathway of tryptophan metabolism, tryptophan 2,3-dioxygenase (TDO2), in multiple types of cancer and its stimulatory role in oncogenic signal transduction through the Kyn pathway [140,141,142]. In a study by T. Miyazaki et al. [143], an analysis of metabolites in colorectal cancer spheroids derived from patients uncovered high levels of Kyn and TDO2, which positively correlated with metastasis to the liver. In the colon cancer spheroids, the TDO2-mediated activation of AhR was found to promote a launch of the Wnt/β-catenin signaling pathway. For instance, an impact of inhibitors of TDO2/AhR on the expression of target genes of Wnt/β-catenin was investigated: *CCND1*, *c-Myc*, *RNF43*, *ZRNF3*, *LGR5*, and *ASCL2*. The inhibition of TDO2 or AhR suppressed all of the tested target genes of Wnt and blocked nuclear localization of β-catenin. An inducible knockout of AhR also repressed the Wnt target genes and the nuclear localization of β-catenin [143]. Among these six Wnt target genes, subsequent in silico analysis showed that promoter regions of the *LGR5* gene contain potential binding sites for AhR, whereas chromatin immunoprecipitation assays confirmed that AhR was bound to the promoter of LGR5 and that this binding was abrogated by inhibitors of AhR or TDO2. Thus, the TDO2–AhR pathway directly modulates the expression of *LGR5*, thereby likely contributing to the upregulation of the Wnt cascade and to the stemness of colon CSCs [143].

##### Downregulation of Wnt Signaling

In several experimental models, toxic AhR ligands have been shown to interfere with Wnt/β-catenin signaling (reviewed by [26,112]).

TCDD

Do interactions between AhR and Wnt/β-catenin pathways participate in the toxic effects of AhR agonists? TCDD is a potent stable ligand of AhR and a well-known toxic substance that enhances tumor formation in various organs [144].

The activation of AhR alters Wnt/β-catenin signaling, thereby impairing tissue regeneration in zebrafish and impeding urogenital-sinus formation during prostate development [112,113]. The activation of AhR by TCDD reduces the levels of Wnt proteins during the early differentiation of mouse embryonic stem cells and inhibits cardiomyocyte functions [145]. The activation of AhR by TCDD has been shown to modulate Wnt/β-catenin signal transduction in WB-F344 rat liver progenitor cells by lowering the levels of the active form of β-catenin and downregulating several target genes of the Wnt/β-catenin pathway [107,146,147].

In liver progenitors, the AhR ligand TCDD reduces the levels of active β-catenin and induces changes in the Wnt pathway such as hypophosphorylation of Dvl, which partakes in the modulation of Wnt signaling [107]. On the other hand, the TCDD-induced weakening of the canonical Wnt pathway in the mouse urogenital sinus has been linked with a decrease in the activity of activators known as R-Spondin2 and R-Spondin3 [145,148].

An analysis of the global profile of gene expression and TCDD-induced changes in the transcriptome of lung carcinoma A549 cells revealed the deregulation of the Wnt/β-catenin signaling pathway in response to TCDD and helped to identify DKK1 (an inhibitor of the Wnt signaling pathway) as a target of AhR [149].

In human HepaRG undifferentiated liver progenitor cells, the TCDD-induced activation of AhR suppresses the expression of target genes of Wnt/β-catenin—*DKK1* and *AXIN2*—the latter of which is a direct transcriptional target of canonical Wnt signaling and may act in a negative feedback loop, thus limiting Wnt signaling [150]. Nonetheless, not all target genes of the Wnt/β-catenin pathway are downregulated by TCDD because the *NKD2* and *MYC* mRNA levels increase in the cells treated with TCDD, indicating a more complicated effect of AhR activation on Wnt signaling than a simple reduction in the β-catenin level and activity [150].

The TCDD-driven activation of AhR significantly inhibits β-catenin expression in mesenchymal stem cells of mice with collagen-induced arthritis [151].

PM2.5

Small particulate air pollution particles smaller than 2.5 µm (PM2.5) are more likely than larger particles to penetrate deep into alveoli, enter the circulation, and activate AhR. It has been shown that AhR activation either by PM2.5 particles or by extractable organic matter (EOM) from PM2.5 can repress Wnt/β-catenin signaling, thereby leading to heart defects in zebrafish embryos [152,153]. Furthermore, the toxicity of PM2.5 to heart development in zebrafish embryos can be prevented by targeting AhR or Wnt/β-catenin signaling. During exposure to PM2.5, an AhR antagonist or β-catenin agonist can restore the normal phenotype of the developing heart [152,154,155,156]. In an article about the P19 cell line (a malignant analog of embryonic stem cells that is able to differentiate toward cardiac lineages), it was reported that the activation of AhR by EOM suppresses the Wnt/β-catenin pathway by reducing the expression of β-catenin, thereby mediating EOM toxicity to heart development and inhibiting cardiac differentiation [157].

FICZ

A direct interaction between the AhR and β-catenin pathways during normal zebrafish embryogenesis is indicated by the finding [158] that AhR2 represses active β-catenin signaling [158]. By means of two AhR agonists having a similar affinity for AhR but a different resistance to metabolic degradation (PCB126 and FICZ) and two β-catenin modulators toxic to zebrafish embryos (XAV939 and AZP), the following was demonstrated. FICZ inhibited the toxicity caused by AZP (increases β-catenin activity by inhibiting GSK3β) and enhanced the toxicity induced by XAV (inhibits β-catenin activity by stabilizing axin). At the same time, the induction of β-catenin–regulated genes (*Axin2*, *Runx2b*, *Rspo2*, and *Pcna*) by AZP was blocked by the presence of FICZ or PCB126 and elevated by an AhR2 knockdown (axin2).

The AhR agonist FICZ blocks the Wnt/β-catenin pathway in human myofibroblasts in an AhR-dependent manner by decreasing the phosphorylation of GSK3β [159]. FICZ inhibits Wnt signaling by diminishing the protein level of β-catenin in intestinal epithelial organoids of C57BL/6 mice [135]. In one report [135], FICZ and TCDD were shown to inhibit in vitro the development of intestinal organoids from mouse crypts or in mouse Lgr5^+^ stem cells. The number of Paneth cells in the small intestine and the depth of the crypts of the small and large intestines were reduced by FICZ administration to mice [135].

I3C

The activation of AhR by a phytochemical ligand called I3C leads to phosphorylation and degradation of β-catenin in prostate cancer DU145 cells [160]. *AhR* expression activated by AhR agonists I3C, IAA, or 3MC lowers the expression of *CTNNB1* in human colon cancer cell lines [108]. AhR as a component of an E3 ubiquitin ligase that is activated by natural ligands derived from indole (which are generated via conversion from dietary tryptophan by intestinal microbes) cleaves β-catenin, thereby inhibiting intestinal carcinogenesis [108]. This is an AhR ligand–dependent pathway of β-catenin degradation that is independent of the APC system.

IS and Kyn

Being AhR agonists that are also tryptophan uremic metabolites, indoxyl sulfate (IS) and Kyn suppress β-catenin in human NIH 3T3 fibroblasts, human epithelial kidney HK-2 cells, and mouse mesenchymal embryonic fibroblasts with an *AhR* knockout by inhibiting the activity of Wnt and proangiogenic targets of Wnt [161]. The just cited study on the mechanism suggests that these uremic substances suppress β-catenin in cells in an AhR-dependent manner based on serine 33 in the degron motif [161].

In another project [162], colon carcinoma HT-29 cells were cultured in a tryptophan-rich DMEM medium. AhR agonist Kyn is the first product of tryptophan breakdown mediated by indolamine 2,3-dioxygenases 1 and 2 (IDO1 or -2) or tryptophan 2,3-dioxygenase (TDO). Furthermore, ref. [162] observed that Kyn promotes goblet cell differentiation of colon carcinoma HT-29 cells by modulating AhR, Wnt, and Notch signals. Those authors demonstrated that Kyn reduces the expression of β-catenin in epithelial cell line HT-29 and that the underexpression of β-catenin was reversed by an IDO-1 inhibitor called 1-methyltryptophan. Those authors hypothesized that Kyn regulates β-catenin expression in an AhR-dependent manner, where AhR acts as a component of a ligand-dependent E3 ubiquitin ligase [162].

Recently, it was found that in neurons, amyloid β activates the IDO1–Kyn–AhR signaling axis, accompanied by the suppression of the Wnt/β-catenin signaling cascade, and that this phenomenon can be reversed by an IDO1 inhibitor [163]. In that work, in primary hippocampal neurons from a Sprague–Dawley rat and hippocampal neurons from an HT22 mouse, the impact of amyloid β on IDO1–Kyn–AhR and Wnt/β-catenin signaling pathways was investigated, as were the reverse effects of IDO1 inhibitors on amyloid-β-induced neuronal damage. It was shown that different concentrations of amyloid β led to a dose-dependent increase in the expression of IDO1 and AhR and phosphorylation of β-catenin. Chromatin immunoprecipitation using cross-linked chromatin from HT22 cells revealed that AhR can bind to XRE sites in the promoter of *DKK1*, which is a negative modulator of Wnt/β-catenin signaling. Thus, the neurotoxicity of amyloid β depends on IDO1–Kyn–AhR signaling. The activation of the IDO1–Kyn–AhR axis through DKK1 suppresses the Wnt/β-catenin pathway, thereby causing neurotoxicity [163].

### 4.2. Wnt Signaling Effects on AhR Signaling

The existence of interactions between Wnt/β-catenin and AhR signaling cascades and the involvement of Wnt/β-catenin signals in the transcriptional control over cytochrome P450 family 1 (CYP1) enzymes are supported by several lines of evidence [26,107,118,164,165,166]. Early research papers have shown that β-catenin interacts with AhR for the induction of CYP1 enzymes. The basal expression of *Cyp1a2* is low in mice with a hepatocyte-specific knockout of *Ctnnb1* [167,168,169], and the induction of CYPs by an AhR agonist called 3MC is weak in this mouse model. The two signaling pathways are synergistic in terms of the induction of AhR target genes by means of TCDD in vitro [107,164,170]. The influence of the WNT/β-catenin pathway on the regulation and inducibility of the main P450 enzymes has been documented by research on the consequences of the treatment of HepaRG cells with an agonist of the WNT/β-catenin signaling pathway (WNT3a) or small interfering RNA (siRNA) against β-catenin, separately or in combination with a set of ligands activating AhR. The evaluation of the expression of P450 genes and P450 enzymatic activity after suppression or activation of the WNT/β-catenin pathway has revealed the necessity of β-catenin, in particular, of AhR-mediated induction of CYP1A [165,171].

#### 4.2.1. The Wnt/β-Catenin Pathway as a Positive Regulator of AhR

An analysis of the currently available data obtained in various rodent liver models and human cell lines suggests that the Wnt/β-catenin pathway is mainly a positive regulator of the expression of AhR and its target genes in many cell types [26,164,172,173]. There is a study about the influence of the chemical nature of AhR agonists (three structurally unrelated agonists of AhR: the polycyclic aromatic hydrocarbon 3MC, the flavonoid βNF, and the antioxidant butylated hydroxyanisole on the interaction of the β-catenin pathway with activated AhR of mice with a hepatocyte-specific knockout of *Ctnnb1* (β-catenin). The study shows that, in mice with the knockout of the β-catenin gene, the attenuation of CYP1 induction does not depend on the chemical nature of an AhR agonist [174].

The transcriptional regulation of *AhR* and of its target genes *CYP1A1* and *CYP1B1* via the WNT/β-catenin pathway is mediated by several molecular mechanisms. First, activated β-catenin may promote AhR overexpression [164,172,173,175]. In particular, a transcriptome analysis has revealed that an overexpressed hyperactive form of β-catenin (featuring a deletion of amino acid residues 24–47) induces *AhR* transcription. Moreover, reporter vectors containing dioxin-sensitive elements are activated by functional β-catenin carrying mutation S33Y [170]. The identification of *AhR* as a target gene for β-catenin has been performed by (1) mimicking the activation of the Wnt signaling cascade by means of β-catenin containing stabilizing mutations, (2) the inhibition of GSK3b by LiCl, and (3) an activator called Wnt3A. Second, the direct transcriptional activation of *CYP1A1* and *CYP1B1* by the β-catenin–TCF transcription complex has been demonstrated. For example, in transgenic mice, the hepatic expression of CYP1A is stimulated by the expression of activated β-catenin (via the S33Y mutation) in the absence of AhR-activating compounds but is suppressed after a β-catenin knockout. These effects were further analyzed in vitro and the stimulatory role of β-catenin was attributed to the TCF-binding site in the promoter of *CYP1A1* [164]. It has been shown that Wnt/β-catenin regulates the transcription of *CYP1B1* in endothelial cells of brain capillaries and in human liver cell lines [176,177]. Third, there is in vitro evidence for the cooperative behavior of β-catenin–TCF and AhR on the human *CYP1A* promoter, where specific binding sites for these transcription factors are located in close proximity [174,178]. Fourth, the activation of β-catenin enhances the transactivation and transcriptional activity of AhR through physical interaction with its XRE in DNA as a coactivator of the AhR–ARNT complex, which induces *Cyp1A1* and *Cyp1A2* [121,164,179,180].

Recently, mechanisms of zonal AhR-mediated induction of *Cyp1A1* under the action of TCDD were determined in a multiscale computational model of a virtual lobule of the mouse liver with embedded stand-alone hepatocytes. It was found that the Wnt/β-catenin signaling cascade maintains basal expression of AhR [181]. The results of the analysis of this model showed that the binding and sequestration of TCDD by CYP1A2, as well as the interaction of transcription factors of the β-catenin gene and the β-catenin–AhR–TCDD complex along with basal metabolic site specificity, determine the zonal induction of *Cyp1A1*.

A regulatory interaction was recently demonstrated between AhR and the Dvl protein, which play a central part in β-catenin stabilization [182]. It was noted that the expression of the AhR protein is substantially dependent on the silencing and overexpression of Dvl in chronic myelogenous leukemia cell lines.

All of the presented data suggest that the Wnt/β-catenin dysregulation associated with an increase in the level or activity of β-catenin may have a positive effect on the expression of CYP1 enzymes participating in the metabolism of procarcinogens, promutagens, natural compounds, endogenous compounds, and some medications.

#### 4.2.2. The Wnt/β-Catenin Pathway as a Negative Regulator of AhR

By contrast, there is an example of a different effect of Wnt/β-catenin signals on AhR signaling: HCT116 human colon cancer cells, where the genotoxicity of BaP has been investigated [183]. In these cells, elevated β-catenin activity due to a mutation of the *CTNNB1* gene plays a key role in the regulation of their growth and tumorigenicity [184]. While evaluating the effect of the inhibition of β-catenin signaling on BaP metabolism and on the expression and activity of the CYP1 enzymes involved in the bioactivation of BaP, those authors discovered that the suppression of β-catenin in an HCT116-based model of colon carcinoma cells carrying an activating mutation of β-catenin enhances the induction of the expression/activity of enzymes CYP1A1, CYP1A2, and CYP1B1 in HCT116 cells by the genotoxic ligand of AhR: BaP [183]. While discussing the discrepancies in the bioactivation of BaP between murine and human models of colonic or intestinal epithelial cells or in the regulation of *CYP1A1* expression between this and some of the above studies, those authors proposed several underlying mechanisms. First, the regulation may be species-specific. Second, there are probably β-catenin–independent effects of the APC protein from the destruction complex (degrading β-catenin), which may contribute to the regulation of CYP1, and there is a truncating *CTNNB1* gene mutation featured by the studied mouse models. Third, different classes of AhR ligands (TCDD, BaP, 3MC, and βNF) may have different requirements for transcription coactivators and may modulate *CYP1* expression in different ways in the context of altered β-catenin signaling [183].

An article by R. Lee et al. [185] about APC-deficient colorectal cancer revealed a relation between WNT signal transduction and the Kyn signaling pathway of tryptophan metabolism, one of the key enzymes of which is **TDO2**. In a synthetic essentiality analysis, TDO2 was identified as a key downstream effector in APC-deficient colorectal cancer. As for the mechanism, a deficiency of APC activated WNT signaling through the upregulation of *TDO2* transcription mediated by transcription complex TCF4/β-catenin. TDO2 in turn activated the Kyn–AhR pathway, which enhanced glycolysis, which stimulates the anabolic growth of cancer cells and CXCL5 secretion to recruit macrophages to the tumor microenvironment. That is, APC deficiency launches the TCF4–TDO2–AhR–CXCL5 cascade, which affects many characteristics of cancer [185].

A recent study about the role of signaling via proliferative and survival pathways, in particular via the Wnt/β-catenin cascade, in the control of *CYP1* expression was undertaken to test the hypothesis that additional indirect mechanisms may participate in the control over *CYP1* expression in tumor cells [186].

In a research project about the regulation of *CYP1* expression in cell models (derived from liver and colon tissues) during the modulation of cell proliferation or the inhibition of transcriptional effectors of proliferative signaling pathways, in particular β-catenin, investigators showed that, in various types of liver cells, cell proliferation is associated with the reduced induction of *CYP1A1*, *CYP1A2*, and *CYP1B1*. That is, the activation of proliferative Wnt/β-catenin signaling leads to the inhibition of *CYP1* expression [186]. Using an siRNA-mediated knockdown of β-catenin, those authors have confirmed that in cells derived from either the human liver or human colon, the suppression of proliferative signaling enhances the induction of CYP1 enzymes by an AhR ligand: TCDD [186].

Different classes of AhR ligands may have different requirements for transcription coactivators, and this state of affairs may result in different types of regulation of *CYP1A1* expression. In a paper about a possible mechanism controlling the expression of AhR-regulated CYP1 enzymes in colon carcinoma HCT-116 cells, it was shown that aberrant proliferative Wnt/β-catenin signaling in tumor cells can attenuate CYP1 induction through competition (with coregulatory protein p300) for the binding to promoters of *CYP1* genes and via the displacement of p300 from the regulatory regions of a *CYP1* gene [186].

#### 4.2.3. Interactions of Wnt and AhR Signaling Cascades in Health and Disease

There are several examples of how the interaction of Wnt/β-catenin and AhR signaling pathways can be important for normal physiology and diseases. For instance, Wnt/β-catenin signaling plays a central role in the establishment and maintenance of a complex blood–brain barrier phenotype in brain endothelial cells, while influencing the metabolic pathways modulated by CYP1B1 [176]. By analyzing the expression of *CYP1B1* in endothelial cells of β-catenin-deficient mice, scientists have demonstrated the necessity of β-catenin for *CYP1B1* expression in cerebrovascular endothelial cells: in the absence of β-catenin, *Cyp1b1* expression was significantly lower [176]. One of functions of CYP1B1 is the production of retinoic acid and an arachidonic acid metabolite (20-hydroxyeicosatetraenoic acid; 20-HETE), which regulate the expression of P-glycoprotein and of junction proteins, respectively. High levels of Cyp1b1 expression can affect the endothelial production of retinoic acid and 20-HETE, thereby promoting vascular homeostasis and the maintenance of the blood–brain barrier. Therefore, the endothelium-specific regulation of *CYP1B1* transcription through the Wnt/β-catenin pathway influences endothelial metabolism and potentially engages CYP1B1 in brain pathologies linked with an impairment of the blood–brain barrier [176].

Another example of the relevance of the interaction between Wnt/β-catenin and AhR signaling pathways for cell physiology and pathologies is the discovery of a new mechanism modulating the expression of *CYP1B1* by means of the TSPYL1 protein via *Wnt/*β-catenin signaling in such disease states as obesity [177]. Lately, members of the gene family called testis-specific Y-encoded-like protein (TSPYL) have been identified as novel transcriptional regulators that may influence the expression of *CYP* genes [187,188], in particular *CYP1B1*, which participates in arachidonic acid metabolism and affects cholesterol concentration. Clinical studies have uncovered elevated plasma and urinary levels of arachidonic acid metabolite 20-HETE in disease states that include obesity [177]. When examining the consequences of the regulation of *CYP1B1* by TSPYL1 and the molecular mechanism by which TSPYL1 regulates the expression of *CYP1B1*, Xiujuan Zhu et al. showed that β-catenin is an activator of *CYP1B1* transcription in HepaRG cells and stimulates cholesterol metabolism with the formation of metabolite 20-HETE. In other words, TSPYL1 is one of the proteins regulating *CYP1B1* expression and having a considerable effect on cholesterol metabolism. This effect of TSPYL1 is implemented by the inhibition of β-catenin binding to TCF/LEF on the *CYP1B1* promoter [177].

## 5. Summary

It can be concluded that AhR has been identified as an inhibitor of canonical Wnt signaling when a specific role has been tested in projects using only a knockout or knockdown of AhR. For instance, a loss of AhR in murine CD11c^+^ intestinal cells is associated with aberrant Wnt signaling caused by the overexpression of target genes *Axin2*, *Lgr5*, *c-Myc*, *Nmp*, and *Dkk3* in intestinal macrophages and the anomalous development of the intestinal epithelium [109]. In intestinal stem cells, AhR modulates the expression of ubiquitin ligases RNF43 and ZNRF3, negatively regulating Wnt/β-catenin signaling and thus limiting cell hyperproliferation, as shown in the AhR^−/−^ mouse model and cultured intestinal organoids [115]. A knockout of AhR enhances Wnt signal transduction in the mouse colon epithelium [116]. The AhR^−/−^ mouse liver phenotype includes faster cell proliferation with insufficient cell polyploidization and the activation of Wnt/β-catenin signaling, possibly owing to AhR being a part of a repressive complex promoting β-catenin ubiquitination and its subsequent degradation [110]. At least coimmunoprecipitation of AhR and β-catenin has been registered under normal conditions in the adult liver [110]. AhR deficiency improves liver regeneration after acute toxic damage by CCl_4_ or after severe mechanical damage to the organ but contributes to the development of hepatocarcinoma, probably in part due to the activation of β-catenin, which is one of the pluripotency factors [111,114]. A knockdown of *AhR* in the murine Schwann cell line MSC80 launches the Wnt/β-catenin pathway by raising the protein level of active β-catenin and the mRNA expression of signaling components Lrp6, Dvl2, Dvl3, and Axin2 by activating TCF/LEF-binding sites [117]. Only one study indicates that AhR signal transduction upregulates the expression of β-catenin and Wnt5a/b, where a knockdown of the *AhR* gene led to the suppression of Wnt5a expression [118]. This was documented in tumor tissue from patients with inflammatory breast cancer demonstrating a constitutive overexpression of AhR.

AhR has been identified as an inhibitor or activator of the canonical Wnt signaling pathway when AhR’s role has been determined in various studies by means of its agonistic ligands such as environmental pollutants (TCDD, PM2.5, 3MC, or BaP), an organophosphate pesticide (CPF), natural compounds (I3C and AS-IV), or endogenous compounds (FICZ, IS, or Kyn).

In some reports, such AhR agonists as TCDD, PM2.5, FICZ, IS, Kyn, and I3C have been shown to impede Wnt/β-catenin signaling [107,135,145,146,147,148,150,151,152,153,154,155,156,157,158,159,161,162,163]. Such data have been obtained in research on zebrafish embryogenesis [158]; the heart of zebrafish embryos [152,153]; mouse embryonic stem cells [145]; rat liver progenitors [107,146,147]; human undifferentiated HepaRG liver progenitors [150]; the P19 cell line; which is a malignant analog of embryonic stem cells [157]; the mouse urogenital sinus [145,148]; lung carcinoma A549 cells [149]; mesenchymal stem cells from mice with collagen-induced arthritis [151]; human myofibroblasts [159]; intestinal epithelial organoids of C57BL/6 mice [135]; human NIH 3T3 fibroblasts, human kidney HK-2 epithelial cells, and mesenchymal embryonic fibroblasts [161]; colon carcinoma HT-29 cells [162]; and mouse hippocampal neuronal HT22 cells [163].

Other studies indicate that similar agonists of AhR, including TCDD, FICZ, Kyn, and I3C, as well as 7,12-dimethylbenz[a]anthracene, Cd, CPF, indeno(1,2,3-cd)pyrene, AS-IV, IAA, and βNF promote the activation of the Wnt/β-catenin signaling pathway [119,120,122,123,124,125,126,127,133,134,136,137,138,143]. Such data have been obtained in research on the chick embryonic thymus [119], the colon carcinoma cell line RKO [120] and other colon cancer cell lines [137], spheroids of colon cancer cells [143], human liver cancer HepG2 cells [122], CSCs from human choriocarcinoma cell line JEG-3 [123], breast cancer MCF-7 and MDA-MB-231 cells [124,126], human ovarian cancer A2780 cells [125], human and murine periodontal ligament cells [127], fetal heart tissues [130], the murine macrophage cell line RAW 264.7 [133], intestinal organoid models derived from intestinal crypts of C57BL/6 mice [134], normal human epidermal melanocytes [136], HCC cell lines HuH-7 and Sk-Hep1 [138], and mouse and human intestinal epithelial cells [139].

The enhancement of canonical Wnt signaling, as follows from the review of the literature, is likely due to the influence of AhR on such upstream components of the Wnt pathway as R-Spondin1 [113], Axin2, Lgr5, and Dkk3 [109]; Lrp6, Dvl2, Dvl3, and Axin2 [117]; Wnt5a/b [118]; and WNT5A, WNT2B, and WNT7B [119]. The enhancement of Wnt/β-catenin signals may be due to the impact of AhR on the expression of β-catenin [118,124,128,130], its activation in the cytoplasm [133,134] owing to greater phosphorylation of AKT and GSK3β [136], and nuclear localization [123,137]. The genes of some upstream components of the Wnt/β-catenin pathway have been shown to be targets of AhR. The promoter regions of genes *CTNNB1*, *RSPO*, *Wnt5a*, *Rnf43*, *LGR*, and *DKK* contain potential binding sites (XREs) for AhR [118,143,149,163,189].

Very recently, a novel AhR signaling mechanism underlying Wnt signal transduction activation was demonstrated [137], in which the SCIN protein, whose expression is regulated by AhR agonists, facilitates nuclear translocation of β-catenin, probably by altering the actin cytoskeleton. The Wnt/β-catenin pathway signals, in turn, are involved in interactions with AhR signaling. An analysis of recent research findings showed that the Wnt/β-catenin pathway is mainly a positive regulator of the expression of AhR and its target genes in various cell types [121,164,174,176,177,179,180,182].

β-catenin plays the main part in the interaction with AhR signaling. Firstly, *AhR* and *CYP1* have been identified as target genes of β-catenin [164,170]; secondly, activated β-catenin may elevate the amount of AhR [164,172,173,175]; thirdly, the binding sites for β-catenin/TCF and AhR in the promoter of *CYP1A* are located in close proximity [174,178]; and, finally, β-catenin interacts with an XRE as a coactivator of the AhR–ARNT complex [121,164,179,180].

Lately, in chronic myeloid leukemia cell lines, a regulatory interaction between AhR and the Dvl protein, which plays a central role in β-catenin stabilization, has been demonstrated; this interaction manifests itself as a dependence of AhR protein expression on the silencing or overexpression of Dvl [182]. There is an example of a different influence on AhR signaling in HCT116 human colon carcinoma cells, where the suppression of β-catenin potentiated the induction of the expression/activity of CYP1 enzymes by BaP [183].

It is worth highlighting the involvement of key enzymes of the Kyn pathway of tryptophan metabolism—TDO2 and IDO1 (which catalyze the formation of Kyn, an agonist of AhR)—in the interactions between AhR and Wnt. The enhancement of the AhR and β-catenin pathways can proceed in a coordinated manner and is a consequence of the activation of the **IDO1** enzyme: after the triggering of IDO1 and the subsequent activation of AhR, β-catenin is activated [138]. The activation of AhR mediated by TDO2 enhances the expression of target genes of Wnt/β-catenin: *CCND1*, *RNF43*, *ZRNF3*, *LGR5*, and *ASCL2* [143].

## 6. Conclusions

Thus, the presented evidence of the influence of AhR pathway signals on the Wnt/catenin signaling cascade paints a complicated picture where the outcome of such an influence is determined by the type of cells, tissues, or organs, as well as by the chemical structure of the AhR ligand. The Wnt/β-catenin pathway signals, in turn, are involved in interactions with AhR signaling. Taken together, the presented literature proves the important role of the interdependent regulation of AhR and Wnt/β-catenin signaling pathways in the maintenance of embryogenesis, organogenesis, and cell homeostasis under normal and pathophysiological conditions. In these interactions of the signaling pathways, AhR appears to act as a fine-tuner of Wnt signal transduction in a cellular, tissue, or organ context; this property indicates the high potential of AhR as a therapeutic target for Wnt pathway modulation in pathological conditions, including cancer and nonmalignant diseases.

## Data Availability

Not applicable.

## References

[1] Hankinson O. (1995). The aryl hydrocarbon receptor complex. Annu. Rev. Pharmacol. Toxicol..

[2] Hankinson O. (2005). Role of coactivators in transcriptional activation by the aryl hydrocarbon receptor. Arch. Biochem. Biophys..

[3] Yin J., Sheng B., Qiu Y., Yang K., Xiao W., Yang H. (2016). Role of AhR in positive regulation of cell proliferation and survival. Cell Prolif..

[4] Sondermann N.C., Fassbender S., Hartung F., Hatala A.M., Rolfes K.M., Vogel C.F.A., Haarmann-Stemmann T. (2023). Functions of the aryl hydrocarbon receptor (AHR) beyond the canonical AHR/ARNT signaling pathway. Biochem. Pharmacol..

[5] Hahn M.E. (2002). Aryl hydrocarbon receptors: Diversity and evolution. Chem. Biol. Interact..

[6] Safe S., Han H., Goldsby J., Mohankumar K., Chapkin R.S. (2018). Aryl Hydrocarbon Receptor (AhR) Ligands as Selective AhR Modulators: Genomic Studies. Curr. Opin. Toxicol..

[7] Esser C., Rannug A. (2015). The aryl hydrocarbon receptor in barrier organ physiology, immunology, and toxicology. Pharmacol. Rev..

[8] Mulero-Navarro S., Fernandez-Salguero P.M. (2016). New Trends in Aryl Hydrocarbon Receptor Biology. Front. Cell Dev. Biol..

[9] Pohjanvirta R. (2012). The AH Receptor in Biology and Toxicology.

[10] Puga A., Ma C., Marlowe J.L. (2009). The aryl hydrocarbon receptor cross-talks with multiple signal transduction pathways. Biochem. Pharmacol..

[11] Larigot L., Benoit L., Koual M., Tomkiewicz C., Barouki R., Coumoul X. (2022). Aryl Hydrocarbon Receptor and Its Diverse Ligands and Functions: An Exposome Receptor. Annu. Rev. Pharmacol. Toxicol..

[12] Wakx A., Nedder M., Tomkiewicz-Raulet C., Dalmasso J., Chissey A., Boland S., Vibert F., Degrelle S.A., Fournier T., Coumoul X. (2018). Expression, Localization, and Activity of the Aryl Hydrocarbon Receptor in the Human Placenta. Int. J. Mol. Sci..

[13] Lee J.S., Cella M., McDonald K.G., Garlanda C., Kennedy G.D., Nukaya M., Mantovani A., Kopan R., Bradfield C.A., Newberry R.D. (2011). AHR drives the development of gut ILC22 cells and postnatal lymphoid tissues via pathways dependent on and independent of Notch. Nat. Immunol..

[14] Roman A.C., Carvajal-Gonzalez J.M., Merino J.M., Mulero-Navarro S., Fernandez-Salguero P.M. (2018). The aryl hydrocarbon receptor in the crossroad of signalling networks with therapeutic value. Pharmacol. Ther..

[15] Serna E., Cespedes C., Vina J. (2020). Anti-Aging Physiological Roles of Aryl Hydrocarbon Receptor and Its Dietary Regulators. Int. J. Mol. Sci..

[16] Sayed T.S., Maayah Z.H., Zeidan H.A., Agouni A., Korashy H.M. (2022). Insight into the physiological and pathological roles of the aryl hydrocarbon receptor pathway in glucose homeostasis, insulin resistance, and diabetes development. Cell Mol. Biol. Lett..

[17] Granados J.C., Falah K., Koo I., Morgan E.W., Perdew G.H., Patterson A.D., Jamshidi N., Nigam S.K. (2022). AHR is a master regulator of diverse pathways in endogenous metabolism. Sci. Rep..

[18] Zablon H.A., Ko C.I., Puga A. (2021). Converging Roles of the Aryl Hydrocarbon Receptor in Early Embryonic Development, Maintenance of Stemness, and Tissue Repair. Toxicol. Sci..

[19] Kou Z., Dai W. (2021). Aryl hydrocarbon receptor: Its roles in physiology. Biochem. Pharmacol..

[20] Hahn M.E., Karchner S.I., Merson R.R. (2017). Diversity as Opportunity: Insights from 600 Million Years of AHR Evolution. Curr. Opin. Toxicol..

[21] Liu J., Xiao Q., Xiao J., Niu C., Li Y., Zhang X., Zhou Z., Shu G., Yin G. (2022). Wnt/beta-catenin signalling: Function, biological mechanisms, and therapeutic opportunities. Signal. Transduct. Target. Ther..

[22] MacDonald B.T., Tamai K., He X. (2009). Wnt/beta-catenin signaling: Components, mechanisms, and diseases. Dev. Cell.

[23] Jung Y.S., Park J.I. (2020). Wnt signaling in cancer: Therapeutic targeting of Wnt signaling beyond beta-catenin and the destruction complex. Exp. Mol. Med..

[24] Lorzadeh S., Kohan L., Ghavami S., Azarpira N. (2021). Autophagy and the Wnt signaling pathway: A focus on Wnt/beta-catenin signaling. Biochim. Biophys. Acta Mol. Cell Res..

[25] Loh K.M., van Amerongen R., Nusse R. (2016). Generating Cellular Diversity and Spatial Form: Wnt Signaling and the Evolution of Multicellular Animals. Dev. Cell.

[26] Schneider A.J., Branam A.M., Peterson R.E. (2014). Intersection of AHR and Wnt signaling in development, health, and disease. Int. J. Mol. Sci..

[27] Nebert D.W. (2017). Aryl hydrocarbon receptor (AHR): "pioneer member" of the basic-helix/loop/helix per-Arnt-sim (bHLH/PAS) family of "sensors" of foreign and endogenous signals. Prog. Lipid Res..

[28] Larigot L., Juricek L., Dairou J., Coumoul X. (2018). AhR signaling pathways and regulatory functions. Biochim. Open.

[29] Ghotbaddini M., Cisse K., Carey A., Powell J.B. (2017). Simultaneous inhibition of aryl hydrocarbon receptor (AhR) and Src abolishes androgen receptor signaling. PLoS ONE.

[30] Bacsi S.G., Hankinson O. (1996). Functional characterization of DNA-binding domains of the subunits of the heterodimeric aryl hydrocarbon receptor complex imputing novel and canonical basic helix-loop-helix protein-DNA interactions. J. Biol. Chem..

[31] Ikuta T., Eguchi H., Tachibana T., Yoneda Y., Kawajiri K. (1998). Nuclear localization and export signals of the human aryl hydrocarbon receptor. J. Biol. Chem..

[32] Beischlag T.V., Morales J.L., Hollingshead B.D., Perdew G.H. (2008). The aryl hydrocarbon receptor complex and the control of gene expression. Crit. Rev. Eukaryot. Gene. Expr..

[33] Tsirpanlis G. (2008). Cellular senescence, cardiovascular risk, and CKD: A review of established and hypothetical interconnections. Am. J. Kidney. Dis..

[34] Matsumura F. (2009). The significance of the nongenomic pathway in mediating inflammatory signaling of the dioxin-activated Ah receptor to cause toxic effects. Biochem. Pharmacol..

[35] Denison M.S., Faber S.C. (2017). And Now for Something Completely Different: Diversity in Ligand-Dependent Activation of Ah Receptor Responses. Curr. Opin. Toxicol..

[36] Wilson S.R., Joshi A.D., Elferink C.J. (2013). The tumor suppressor Kruppel-like factor 6 is a novel aryl hydrocarbon receptor DNA binding partner. J. Pharmacol. Exp. Ther..

[37] Ge N.L., Elferink C.J. (1998). A direct interaction between the aryl hydrocarbon receptor and retinoblastoma protein. Linking dioxin signaling to the cell cycle. J. Biol. Chem..

[38] Puga A., Barnes S.J., Dalton T.P., Chang C., Knudsen E.S., Maier M.A. (2000). Aromatic hydrocarbon receptor interaction with the retinoblastoma protein potentiates repression of E2F-dependent transcription and cell cycle arrest. J. Biol. Chem..

[39] Marlowe J.L., Knudsen E.S., Schwemberger S., Puga A. (2004). The aryl hydrocarbon receptor displaces p300 from E2F-dependent promoters and represses S phase-specific gene expression. J. Biol. Chem..

[40] Grishanova A.Y., Perepechaeva M.L. (2022). Aryl Hydrocarbon Receptor in Oxidative Stress as a Double Agent and Its Biological and Therapeutic Significance. Int. J. Mol. Sci..

[41] Ishihara Y., Kado S.Y., Hoeper C., Harel S., Vogel C.F.A. (2019). Role of NF-kB RelB in Aryl Hydrocarbon Receptor-Mediated Ligand Specific Effects. Int. J. Mol. Sci..

[42] Kim D.W., Gazourian L., Quadri S.A., Romieu-Mourez R., Sherr D.H., Sonenshein G.E. (2000). The RelA NF-kappaB subunit and the aryl hydrocarbon receptor (AhR) cooperate to transactivate the c-myc promoter in mammary cells. Oncogene.

[43] Kimura A., Naka T., Nohara K., Fujii-Kuriyama Y., Kishimoto T. (2008). Aryl hydrocarbon receptor regulates Stat1 activation and participates in the development of Th17 cells. Proc. Natl. Acad. Sci. USA.

[44] Szelest M., Walczak K., Plech T. (2021). A New Insight into the Potential Role of Tryptophan-Derived AhR Ligands in Skin Physiological and Pathological Processes. Int. J. Mol. Sci..

[45] Lamas B., Natividad J.M., Sokol H. (2018). Aryl hydrocarbon receptor and intestinal immunity. Mucosal. Immunol..

[46] Salminen A. (2022). Aryl hydrocarbon receptor (AhR) reveals evidence of antagonistic pleiotropy in the regulation of the aging process. Cell Mol. Life Sci..

[47] Jackson D.P., Li H., Mitchell K.A., Joshi A.D., Elferink C.J. (2014). Ah receptor-mediated suppression of liver regeneration through NC-XRE-driven p21Cip1 expression. Mol. Pharmacol..

[48] Bock K.W. (2019). Aryl hydrocarbon receptor (AHR): From selected human target genes and crosstalk with transcription factors to multiple AHR functions. Biochem. Pharmacol..

[49] Lee W.J., Liu S.H., Chiang C.K., Lin S.Y., Liang K.W., Chen C.H., Tien H.R., Chen P.H., Wu J.P., Tsai Y.C. (2016). Aryl Hydrocarbon Receptor Deficiency Attenuates Oxidative Stress-Related Mesangial Cell Activation and Macrophage Infiltration and Extracellular Matrix Accumulation in Diabetic Nephropathy. Antioxid. Redox. Signal..

[50] Zangar R.C., Davydov D.R., Verma S. (2004). Mechanisms that regulate production of reactive oxygen species by cytochrome P450. Toxicol. Appl. Pharmacol..

[51] Poland A., Palen D., Glover E. (1982). Tumour promotion by TCDD in skin of HRS/J hairless mice. Nature.

[52] Duarte-Hospital C., Tete A., Brial F., Benoit L., Koual M., Tomkiewicz C., Kim M.J., Blanc E.B., Coumoul X., Bortoli S. (2021). Mitochondrial Dysfunction as a Hallmark of Environmental Injury. Cells.

[53] Gearhart-Serna L.M., Davis J.B., Jolly M.K., Jayasundara N., Sauer S.J., Di Giulio R.T., Devi G.R. (2020). A polycyclic aromatic hydrocarbon-enriched environmental chemical mixture enhances AhR, antiapoptotic signaling and a proliferative phenotype in breast cancer cells. Carcinogenesis.

[54] Randi A.S., Sanchez M.S., Alvarez L., Cardozo J., Pontillo C., de Pisarev D.L.K. (2008). Hexachlorobenzene triggers AhR translocation to the nucleus, c-Src activation and EGFR transactivation in rat liver. Toxicol. Lett..

[55] Oda K., Matsuoka Y., Funahashi A., Kitano H. (2005). A comprehensive pathway map of epidermal growth factor receptor signaling. Mol. Syst. Biol..

[56] Tomkiewicz C., Herry L., Bui L.C., Metayer C., Bourdeloux M., Barouki R., Coumoul X. (2013). The aryl hydrocarbon receptor regulates focal adhesion sites through a non-genomic FAK/Src pathway. Oncogene.

[57] Xie G., Peng Z., Raufman J.P. (2012). Src-mediated aryl hydrocarbon and epidermal growth factor receptor cross talk stimulates colon cancer cell proliferation. Am. J. Physiol. Gastrointest. Liver Physiol..

[58] Ohtake F., Baba A., Takada I., Okada M., Iwasaki K., Miki H., Takahashi S., Kouzmenko A., Nohara K., Chiba T. (2007). Dioxin receptor is a ligand-dependent E3 ubiquitin ligase. Nature.

[59] Luecke-Johansson S., Gralla M., Rundqvist H., Ho J.C., Johnson R.S., Gradin K., Poellinger L. (2017). A Molecular Mechanism To Switch the Aryl Hydrocarbon Receptor from a Transcription Factor to an E3 Ubiquitin Ligase. Mol. Cell Biol..

[60] Denison M.S., Nagy S.R. (2003). Activation of the aryl hydrocarbon receptor by structurally diverse exogenous and endogenous chemicals. Annu. Rev. Pharmacol. Toxicol..

[61] Avilla M.N., Malecki K.M.C., Hahn M.E., Wilson R.H., Bradfield C.A. (2020). The Ah Receptor: Adaptive Metabolism, Ligand Diversity, and the Xenokine Model. Chem. Res. Toxicol..

[62] Denison M.S., Pandini A., Nagy S.R., Baldwin E.P., Bonati L. (2002). Ligand binding and activation of the Ah receptor. Chem. Biol. Interact..

[63] Nguyen L.P., Bradfield C.A. (2008). The search for endogenous activators of the aryl hydrocarbon receptor. Chem. Res. Toxicol..

[64] Ronnekleiv-Kelly S.M., Nukaya M., Diaz-Diaz C.J., Megna B.W., Carney P.R., Geiger P.G., Kennedy G.D. (2016). Aryl hydrocarbon receptor-dependent apoptotic cell death induced by the flavonoid chrysin in human colorectal cancer cells. Cancer Lett..

[65] Ikuta T., Kurosumi M., Yatsuoka T., Nishimura Y. (2016). Tissue distribution of aryl hydrocarbon receptor in the intestine: Implication of putative roles in tumor suppression. Exp. Cell Res..

[66] Pastorkova B., Vrzalova A., Bachleda P., Dvorak Z. (2017). Hydroxystilbenes and methoxystilbenes activate human aryl hydrocarbon receptor and induce CYP1A genes in human hepatoma cells and human hepatocytes. Food Chem. Toxicol..

[67] Safe S., Lee S.O., Jin U.H. (2013). Role of the aryl hydrocarbon receptor in carcinogenesis and potential as a drug target. Toxicol. Sci..

[68] Petriello M.C., Hoffman J.B., Morris A.J., Hennig B. (2017). Emerging roles of xenobiotic detoxification enzymes in metabolic diseases. Rev. Environ. Health.

[69] Rejano-Gordillo C., Ordiales-Talavero A., Nacarino-Palma A., Merino J.M., Gonzalez-Rico F.J., Fernandez-Salguero P.M. (2022). Aryl Hydrocarbon Receptor: From Homeostasis to Tumor Progression. Front. Cell Dev. Biol..

[70] Rejano-Gordillo C.M., Marin-Diaz B., Ordiales-Talavero A., Merino J.M., Gonzalez-Rico F.J., Fernandez-Salguero P.M. (2022). From Nucleus to Organs: Insights of Aryl Hydrocarbon Receptor Molecular Mechanisms. Int. J. Mol. Sci..

[71] Busbee P.B., Rouse M., Nagarkatti M., Nagarkatti P.S. (2013). Use of natural AhR ligands as potential therapeutic modalities against inflammatory disorders. Nutr. Rev..

[72] Backlund M., Ingelman-Sundberg M. (2004). Different structural requirements of the ligand binding domain of the aryl hydrocarbon receptor for high- and low-affinity ligand binding and receptor activation. Mol. Pharmacol..

[73] Guyot E., Chevallier A., Barouki R., Coumoul X. (2013). The AhR twist: Ligand-dependent AhR signaling and pharmaco-toxicological implications. Drug Discov. Today.

[74] Ciolino H.P., Daschner P.J., Yeh G.C. (1999). Dietary flavonols quercetin and kaempferol are ligands of the aryl hydrocarbon receptor that affect CYP1A1 transcription differentially. Biochem. J..

[75] Hubbard T.D., Murray I.A., Bisson W.H., Lahoti T.S., Gowda K., Amin S.G., Patterson A.D., Perdew G.H. (2015). Adaptation of the human aryl hydrocarbon receptor to sense microbiota-derived indoles. Sci. Rep..

[76] Phelan D., Winter G.M., Rogers W.J., Lam J.C., Denison M.S. (1998). Activation of the Ah receptor signal transduction pathway by bilirubin and biliverdin. Arch. Biochem. Biophys..

[77] Adachi J., Mori Y., Matsui S., Takigami H., Fujino J., Kitagawa H., Miller C.A., Kato T., Saeki K., Matsuda T. (2001). Indirubin and indigo are potent aryl hydrocarbon receptor ligands present in human urine. J. Biol. Chem..

[78] Guengerich F.P., Martin M.V., McCormick W.A., Nguyen L.P., Glover E., Bradfield C.A. (2004). Aryl hydrocarbon receptor response to indigoids in vitro and in vivo. Arch. Biochem. Biophys..

[79] Collins S.L., Patterson A.D. (2020). The gut microbiome: An orchestrator of xenobiotic metabolism. Acta Pharm. Sin. B.

[80] Jin U.H., Lee S.O., Sridharan G., Lee K., Davidson L.A., Jayaraman A., Chapkin R.S., Alaniz R., Safe S. (2014). Microbiome-derived tryptophan metabolites and their aryl hydrocarbon receptor-dependent agonist and antagonist activities. Mol. Pharmacol..

[81] Miller C.A. (1999). A human aryl hydrocarbon receptor signaling pathway constructed in yeast displays additive responses to ligand mixtures. Toxicol. Appl. Pharmacol..

[82] Moura-Alves P., Fae K., Houthuys E., Dorhoi A., Kreuchwig A., Furkert J., Barison N., Diehl A., Munder A., Constant P. (2014). AhR sensing of bacterial pigments regulates antibacterial defence. Nature.

[83] Denison M.S., Soshilov A.A., He G., DeGroot D.E., Zhao B. (2011). Exactly the same but different: Promiscuity and diversity in the molecular mechanisms of action of the aryl hydrocarbon (dioxin) receptor. Toxicol. Sci..

[84] Komiya Y., Habas R. (2008). Wnt signal transduction pathways. Organogenesis.

[85] Moon R.T., Kohn A.D., De Ferrari G.V., Kaykas A. (2004). WNT and beta-catenin signalling: Diseases and therapies. Nat. Rev. Genet..

[86] Linding R., Jensen L.J., Ostheimer G.J., van Vugt M.A., Jorgensen C., Miron I.M., Diella F., Colwill K., Taylor L., Elder K. (2007). Systematic discovery of in vivo phosphorylation networks. Cell.

[87] Yang Y., Chan W.K. (2021). Glycogen Synthase Kinase 3 Beta Regulates the Human Aryl Hydrocarbon Receptor Cellular Content and Activity. Int. J. Mol. Sci..

[88] Albanese I., Khan K., Barratt B., Al-Kindi H., Schwertani A. (2018). Atherosclerotic Calcification: Wnt Is the Hint. J. Am. Heart Assoc..

[89] Nusse R., Clevers H. (2017). Wnt/beta-Catenin Signaling, Disease, and Emerging Therapeutic Modalities. Cell.

[90] Niehrs C. (2012). The complex world of WNT receptor signalling. Nat. Rev. Mol. Cell Biol..

[91] Schaefer K.N., Pronobis M.I., Williams C.E., Zhang S., Bauer L., Goldfarb D., Yan F., Major M.B., Peifer M. (2020). Wnt regulation: Exploring Axin-Disheveled interactions and defining mechanisms by which the SCF E3 ubiquitin ligase is recruited to the destruction complex. Mol. Biol. Cell.

[92] Gao C., Chen Y.G. (2010). Dishevelled: The hub of Wnt signaling. Cell Signal..

[93] Kafka A., Basic-Kinda S., Pecina-Slaus N. (2014). The cellular story of dishevelleds. Croat. Med. J..

[94] Bian J., Dannappel M., Wan C., Firestein R. (2020). Transcriptional Regulation of Wnt/beta-Catenin Pathway in Colorectal Cancer. Cells.

[95] Schuijers J., Mokry M., Hatzis P., Cuppen E., Clevers H. (2014). Wnt-induced transcriptional activation is exclusively mediated by TCF/LEF. EMBO J..

[96] Torres V.I., Godoy J.A., Inestrosa N.C. (2019). Modulating Wnt signaling at the root: Porcupine and Wnt acylation. Pharmacol. Ther..

[97] Kikuchi A., Matsumoto S., Sada R. (2022). Dickkopf signaling, beyond Wnt-mediated biology. Semin. Cell Dev. Biol..

[98] Chien A.J., Conrad W.H., Moon R.T. (2009). A Wnt survival guide: From flies to human disease. J. Investig. Dermatol..

[99] Niida A., Hiroko T., Kasai M., Furukawa Y., Nakamura Y., Suzuki Y., Sugano S., Akiyama T. (2004). DKK1, a negative regulator of Wnt signaling, is a target of the beta-catenin/TCF pathway. Oncogene.

[100] Hao H.X., Jiang X., Cong F. (2016). Control of Wnt Receptor Turnover by R-spondin-ZNRF3/RNF43 Signaling Module and Its Dysregulation in Cancer. Cancers.

[101] Hao H.X., Xie Y., Zhang Y., Charlat O., Oster E., Avello M., Lei H., Mickanin C., Liu D., Ruffner H. (2012). ZNRF3 promotes Wnt receptor turnover in an R-spondin-sensitive manner. Nature.

[102] de Lau W., Barker N., Low T.Y., Koo B.K., Li V.S., Teunissen H., Kujala P., Haegebarth A., Peters P.J., van de Wetering M. (2011). Lgr5 homologues associate with Wnt receptors and mediate R-spondin signalling. Nature.

[103] Giannakis M., Hodis E., Mu X.J., Yamauchi M., Rosenbluh J., Cibulskis K., Saksena G., Lawrence M.S., Qian Z.R., Nishihara R. (2014). RNF43 is frequently mutated in colorectal and endometrial cancers. Nat. Genet..

[104] Ter Steege E.J., Bakker E.R.M. (2021). The role of R-spondin proteins in cancer biology. Oncogene.

[105] Nusse R. The Wnt Homepage. http://web.stanford.edu/group/nusselab/cgi-bin/wnt/.

[106] Katoh M., Katoh M. (2017). Molecular genetics and targeted therapy of WNT-related human diseases (Review). Int. J. Mol. Med..

[107] Prochazkova J., Kabatkova M., Bryja V., Umannova L., Bernatik O., Kozubik A., Machala M., Vondracek J. (2011). The interplay of the aryl hydrocarbon receptor and beta-catenin alters both AhR-dependent transcription and Wnt/beta-catenin signaling in liver progenitors. Toxicol. Sci..

[108] Kawajiri K., Kobayashi Y., Ohtake F., Ikuta T., Matsushima Y., Mimura J., Pettersson S., Pollenz R.S., Sakaki T., Hirokawa T. (2009). Aryl hydrocarbon receptor suppresses intestinal carcinogenesis in *Apc*^*Min*/+^ mice with natural ligands. Proc. Natl. Acad. Sci. USA.

[109] Chng S.H., Kundu P., Dominguez-Brauer C., Teo W.L., Kawajiri K., Fujii-Kuriyama Y., Mak T.W., Pettersson S. (2016). Ablating the aryl hydrocarbon receptor (AhR) in CD11c+ cells perturbs intestinal epithelium development and intestinal immunity. Sci. Rep..

[110] Moreno-Marin N., Merino J.M., Alvarez-Barrientos A., Patel D.P., Takahashi S., Gonzalez-Sancho J.M., Gandolfo P., Rios R.M., Munoz A., Gonzalez F.J. (2018). Aryl Hydrocarbon Receptor Promotes Liver Polyploidization and Inhibits PI3K, ERK, and Wnt/beta-Catenin Signaling. iScience.

[111] Moreno-Marin N., Barrasa E., Morales-Hernandez A., Paniagua B., Blanco-Fernandez G., Merino J.M., Fernandez-Salguero P.M. (2017). Dioxin Receptor Adjusts Liver Regeneration After Acute Toxic Injury and Protects Against Liver Carcinogenesis. Sci. Rep..

[112] Mathew L.K., Simonich M.T., Tanguay R.L. (2009). AHR-dependent misregulation of Wnt signaling disrupts tissue regeneration. Biochem. Pharmacol..

[113] Mathew L.K., Sengupta S.S., Ladu J., Andreasen E.A., Tanguay R.L. (2008). Crosstalk between AHR and Wnt signaling through R-Spondin1 impairs tissue regeneration in zebrafish. FASEB J..

[114] Rejano-Gordillo C.M., Gonzalez-Rico F.J., Marin-Diaz B., Ordiales-Talavero A., Nacarino-Palma A., Roman A.C., Merino J.M., Fernandez-Salguero P.M. (2022). Liver regeneration after partial hepatectomy is improved in the absence of aryl hydrocarbon receptor. Sci. Rep..

[115] Metidji A., Omenetti S., Crotta S., Li Y., Nye E., Ross E., Li V., Maradana M.R., Schiering C., Stockinger B. (2018). The Environmental Sensor AHR Protects from Inflammatory Damage by Maintaining Intestinal Stem Cell Homeostasis and Barrier Integrity. Immunity.

[116] Han H., Davidson L.A., Hensel M., Yoon G., Landrock K., Allred C., Jayaraman A., Ivanov I., Safe S.H., Chapkin R.S. (2021). Loss of Aryl Hydrocarbon Receptor Promotes Colon Tumorigenesis in *Apc*^*S580*/+^; *Kras*^*G12D*/+^ Mice. Mol. Cancer Res..

[117] Shackleford G., Sampathkumar N.K., Hichor M., Weill L., Meffre D., Juricek L., Laurendeau I., Chevallier A., Ortonne N., Larousserie F. (2018). Involvement of Aryl hydrocarbon receptor in myelination and in human nerve sheath tumorigenesis. Proc. Natl. Acad. Sci. USA.

[118] Mohamed H.T., Gadalla R., El-Husseiny N., Hassan H., Wang Z., Ibrahim S.A., El-Shinawi M., Sherr D.H., Mohamed M.M. (2019). Inflammatory breast cancer: Activation of the aryl hydrocarbon receptor and its target CYP1B1 correlates closely with Wnt5a/b-beta-catenin signalling, the stem cell phenotype and disease progression. J. Adv. Res..

[119] Cho M.K., Park J.G., Iwata H., Kim E.Y. (2021). 2,3,7,8-Tetrachlorodibenzo-p-dioxin prompted differentiation to CD4^+^CD8^−^CD25^+^ and CD4^+^CD8^+^CD25^+^ Tregs and altered expression of immune-related genes in the thymus of chicken embryos. Ecotoxicol. Environ. Saf..

[120] Yamaguchi M., Hankinson O. (2019). 2,3,7,8-tetrachlorodibenzo-p-dioxin suppresses the growth of human colorectal cancer cells in vitro: Implication of the aryl hydrocarbon receptor signaling. Int. J. Oncol..

[121] Shiizaki K., Kido K., Mizuta Y. (2019). Insight into the relationship between aryl-hydrocarbon receptor and beta-catenin in human colon cancer cells. PLoS ONE.

[122] Yamaguchi M., Hankinson O. (2018). 2,3,7,8-Tetrachlorodibenzo-p-dioxin suppresses the growth of human liver cancer HepG2 cells in vitro: Involvement of cell signaling factors. Int. J. Oncol..

[123] Wu C., Yu S., Tan Q., Guo P., Liu H. (2018). Role of AhR in regulating cancer stem cell-like characteristics in choriocarcinoma. Cell Cycle.

[124] Al-Dhfyan A., Alhoshani A., Korashy H.M. (2017). Aryl hydrocarbon receptor/cytochrome P450 1A1 pathway mediates breast cancer stem cells expansion through PTEN inhibition and beta-Catenin and Akt activation. Mol. Cancer.

[125] Therachiyil L., Krishnankutty R., Ahmad F., Mateo J.M., Uddin S., Korashy H.M. (2022). Aryl Hydrocarbon Receptor Promotes Cell Growth, Stemness Like Characteristics, and Metastasis in Human Ovarian Cancer via Activation of PI3K/Akt, beta-Catenin, and Epithelial to Mesenchymal Transition Pathways. Int. J. Mol. Sci..

[126] Moyano P., Garcia J.M., Garcia J., Pelayo A., Munoz-Calero P., Frejo M.T., Flores A., Del Pino J. (2021). Aryl Hydrocarbon Receptor Activation Produces Heat Shock Protein 90 and 70 Overexpression, Prostaglandin E2/Wnt/beta-Catenin Signaling Disruption, and Cell Proliferation in MCF-7 and MDA-MB-231 Cells after 24 h and 14 Days of Chlorpyrifos Treatment. Chem. Res. Toxicol..

[127] Huang J., Cai X., Ou Y., Fan L., Zhou Y., Wang Y. (2019). Protective roles of FICZ and aryl hydrocarbon receptor axis on alveolar bone loss and inflammation in experimental periodontitis. J. Clin. Periodontol..

[128] Keshavarzi M., Khoshnoud M.J., Bahraman A.G., Mohammadi-Bardbori A. (2020). An Endogenous Ligand of Aryl Hydrocarbon Receptor 6-Formylindolo[3,2-b]Carbazole (FICZ) Is a Signaling Molecule in Neurogenesis of Adult Hippocampal Neurons. J. Mol. Neurosci..

[129] Keshavarzi M., Moradbeygi F., Mobini K., Bahraman A.G., Mohammadi P., Ghaedi A., Mohammadi-Bardbori A. (2022). The interplay of aryl hydrocarbon receptor/WNT/CTNNB1/Notch signaling pathways regulate amyloid beta precursor mRNA/protein expression and effected the learning and memory of mice. Toxicol. Res..

[130] Omidi M., Niknahad H., Noorafshan A., Fardid R., Nadimi E., Naderi S., Bakhtari A., Mohammadi-Bardbori A. (2019). Co-exposure to an Aryl Hydrocarbon Receptor Endogenous Ligand, 6-Formylindolo[3,2-b]carbazole (FICZ), and Cadmium Induces Cardiovascular Developmental Abnormalities in Mice. Biol. Trace Elem. Res..

[131] Shivanna B., Chu C., Moorthy B. (2022). The Aryl Hydrocarbon Receptor (AHR): A Novel Therapeutic Target for Pulmonary Diseases?. Int. J. Mol. Sci..

[132] Takei H., Yasuoka H., Yoshimoto K., Takeuchi T. (2020). Aryl hydrocarbon receptor signals attenuate lung fibrosis in the bleomycin-induced mouse model for pulmonary fibrosis through increase of regulatory T cells. Arthritis. Res. Ther..

[133] Selvam P., Cheng C.M., Dahms H.U., Ponnusamy V.K., Sun Y.Y. (2022). AhR Mediated Activation of Pro-Inflammatory Response of RAW 264.7 Cells Modulate the Epithelial-Mesenchymal Transition. Toxics.

[134] Park J.H., Lee J.M., Lee E.J., Hwang W.B., Kim D.J. (2018). Indole-3-Carbinol Promotes Goblet-Cell Differentiation Regulating Wnt and Notch Signaling Pathways AhR-Dependently. Mol. Cells.

[135] Park J.H., Choi A.J., Kim S.J., Cheong S.W., Jeong S.Y. (2016). AhR activation by 6-formylindolo[3,2-b]carbazole and 2,3,7,8-tetrachlorodibenzo-p-dioxin inhibit the development of mouse intestinal epithelial cells. Environ. Toxicol. Pharmacol..

[136] Liu B., Xie Y., Wu Z. (2020). Astragaloside IV Enhances Melanogenesis via the AhR-Dependent AKT/GSK-3beta/beta-Catenin Pathway in Normal Human Epidermal Melanocytes. Evid. Based. Complement. Alternat. Med..

[137] Perez-Castro L., Venkateswaran N., Garcia R., Hao Y.H., Lafita-Navarro M.C., Kim J., Segal D., Saponzik E., Chang B.J., Fiolka R. (2022). The AHR target gene scinderin activates the WNT pathway by facilitating the nuclear translocation of beta-catenin. J. Cell Sci..

[138] Chen C.T., Wu P.H., Hu C.C., Nien H.C., Wang J.T., Sheu J.C., Chow L.P. (2021). Aberrant Upregulation of Indoleamine 2,3-Dioxygenase 1 Promotes Proliferation and Metastasis of Hepatocellular Carcinoma Cells via Coordinated Activation of AhR and beta-Catenin Signaling. Int. J. Mol. Sci..

[139] Bishnupuri K.S., Alvarado D.M., Khouri A.N., Shabsovich M., Chen B., Dieckgraefe B.K., Ciorba M.A. (2019). IDO1 and Kynurenine Pathway Metabolites Activate PI3K-Akt Signaling in the Neoplastic Colon Epithelium to Promote Cancer Cell Proliferation and Inhibit Apoptosis. Cancer Res..

[140] D’Amato N.C., Rogers T.J., Gordon M.A., Greene L.I., Cochrane D.R., Spoelstra N.S., Nemkov T.G., D’Alessandro A., Hansen K.C., Richer J.K. (2015). A TDO2-AhR signaling axis facilitates anoikis resistance and metastasis in triple-negative breast cancer. Cancer Res..

[141] van Baren N., Van den Eynde B.J. (2015). Tryptophan-degrading enzymes in tumoral immune resistance. Front. Immunol..

[142] Ott M., Litzenburger U.M., Rauschenbach K.J., Bunse L., Ochs K., Sahm F., Pusch S., Opitz C.A., Blaes J., von Deimling A. (2015). Suppression of TDO-mediated tryptophan catabolism in glioblastoma cells by a steroid-responsive FKBP52-dependent pathway. Glia.

[143] Miyazaki T., Chung S., Sakai H., Ohata H., Obata Y., Shiokawa D., Mizoguchi Y., Kubo T., Ichikawa H., Taniguchi H. (2022). Stemness and immune evasion conferred by the TDO2-AHR pathway are associated with liver metastasis of colon cancer. Cancer Sci..

[144] Knerr S., Schrenk D. (2006). Carcinogenicity of 2,3,7,8-tetrachlorodibenzo-p-dioxin in experimental models. Mol. Nutr. Food Res..

[145] Wang Q., Kurita H., Carreira V., Ko C.I., Fan Y., Zhang X., Biesiada J., Medvedovic M., Puga A. (2016). Ah Receptor Activation by Dioxin Disrupts Activin, BMP, and WNT Signals During the Early Differentiation of Mouse Embryonic Stem Cells and Inhibits Cardiomyocyte Functions. Toxicol. Sci..

[146] Faust D., Vondracek J., Krcmar P., Smerdova L., Prochazkova J., Hruba E., Hulinkova P., Kaina B., Dietrich C., Machala M. (2013). AhR-mediated changes in global gene expression in rat liver progenitor cells. Arch. Toxicol..

[147] Svobodova J., Kabatkova M., Smerdova L., Brenerova P., Dvorak Z., Machala M., Vondracek J. (2015). The aryl hydrocarbon receptor-dependent disruption of contact inhibition in rat liver WB-F344 epithelial cells is linked with induction of survivin, but not with inhibition of apoptosis. Toxicology.

[148] Branam A.M., Davis N.M., Moore R.W., Schneider A.J., Vezina C.M., Peterson R.E. (2013). TCDD inhibition of canonical Wnt signaling disrupts prostatic bud formation in mouse urogenital sinus. Toxicol. Sci..

[149] Prochazkova J., Strapacova S., Svrzkova L., Andrysik Z., Hyzdalova M., Hruba E., Pencikova K., Libalova H., Topinka J., Klema J. (2018). Adaptive changes in global gene expression profile of lung carcinoma A549 cells acutely exposed to distinct types of AhR ligands. Toxicol. Lett..

[150] Svobodova J., Prochazkova J., Kabatkova M., Krkoska M., Smerdova L., Libalova H., Topinka J., Klema J., Kozubik A., Machala M. (2019). 2,3,7,8-Tetrachlorodibenzo-p-dioxin (TCDD) Disrupts Control of Cell Proliferation and Apoptosis in a Human Model of Adult Liver Progenitors. Toxicol. Sci..

[151] Tong Y., Niu M., Du Y., Mei W., Cao W., Dou Y., Yu H., Du X., Yuan H., Zhao W. (2017). Aryl hydrocarbon receptor suppresses the osteogenesis of mesenchymal stem cells in collagen-induced arthritic mice through the inhibition of beta-catenin. Exp. Cell Res..

[152] Zhang H., Yao Y., Chen Y., Yue C., Chen J., Tong J., Jiang Y., Chen T. (2016). Crosstalk between AhR and wnt/beta-catenin signal pathways in the cardiac developmental toxicity of PM2.5 in zebrafish embryos. Toxicology.

[153] Zhang M., Chen J., Jiang Y., Chen T. (2022). Fine particulate matter induces heart defects via AHR/ROS-mediated endoplasmic reticulum stress. Chemosphere.

[154] Massarsky A., Bone A.J., Dong W., Hinton D.E., Prasad G.L., Di Giulio R.T. (2016). AHR2 morpholino knockdown reduces the toxicity of total particulate matter to zebrafish embryos. Toxicol. Appl. Pharmacol..

[155] Yue C., Ji C., Zhang H., Zhang L.W., Tong J., Jiang Y., Chen T. (2017). Protective effects of folic acid on PM2.5-induced cardiac developmental toxicity in zebrafish embryos by targeting AhR and Wnt/beta-catenin signal pathways. Environ. Toxicol..

[156] Chen J., Zhang M., Zou H., Aniagu S., Jiang Y., Chen T. (2022). Synergistic protective effects of folic acid and resveratrol against fine particulate matter-induced heart malformations in zebrafish embryos. Ecotoxicol. Environ. Saf..

[157] Chen T., Jin H., Wang H., Yao Y., Aniagu S., Tong J., Jiang Y. (2019). Aryl hydrocarbon receptor mediates the cardiac developmental toxicity of EOM from PM (2.5) in P19 embryonic carcinoma cells. Chemosphere.

[158] Wincent E., Stegeman J.J., Jonsson M.E. (2015). Combination effects of AHR agonists and Wnt/beta-catenin modulators in zebrafish embryos: Implications for physiological and toxicological AHR functions. Toxicol. Appl. Pharmacol..

[159] Woeller C.F., Roztocil E., Hammond C.L., Feldon S.E., Phipps R.P. (2016). The Aryl Hydrocarbon Receptor and Its Ligands Inhibit Myofibroblast Formation and Activation: Implications for Thyroid Eye Disease. Am. J. Pathol..

[160] Jeong Y.M., Li H., Kim S.Y., Yun H.Y., Baek K.J., Kwon N.S., Myung S.C., Kim D.S. (2011). Indole-3-carbinol inhibits prostate cancer cell migration via degradation of beta-catenin. Oncol. Res..

[161] Arinze N.V., Yin W., Lotfollahzadeh S., Napoleon M.A., Richards S., Walker J.A., Belghasem M., Ravid J.D., Kamel M.H., Whelan S.A. (2022). Tryptophan metabolites suppress the Wnt pathway and promote adverse limb events in chronic kidney disease. J. Clin. Investig..

[162] Park J.H., Lee J.M., Lee E.J., Kim D.J., Hwang W.B. (2018). Kynurenine promotes the goblet cell differentiation of HT-29 colon carcinoma cells by modulating Wnt, Notch and AhR signals. Oncol. Rep..

[163] Duan Z., Zhang S., Liang H., Xing Z., Guo L., Shi L., Du L., Kuang C., Takikawa O., Yang Q. (2020). Amyloid beta neurotoxicity is IDO1-Kyn-AhR dependent and blocked by IDO1 inhibitor. Signal. Transduct. Target. Ther..

[164] Braeuning A., Kohle C., Buchmann A., Schwarz M. (2011). Coordinate regulation of cytochrome P450 1a1 expression in mouse liver by the aryl hydrocarbon receptor and the beta-catenin pathway. Toxicol. Sci..

[165] Thomas M., Bayha C., Vetter S., Hofmann U., Schwarz M., Zanger U.M., Braeuning A. (2015). Activating and Inhibitory Functions of WNT/beta-Catenin in the Induction of Cytochromes P450 by Nuclear Receptors in HepaRG Cells. Mol. Pharmacol..

[166] Braeuning A., Schwarz M. (2020). Regulation of expression of drug-metabolizing enzymes by oncogenic signaling pathways in liver tumors: A review. Acta Pharm. Sin. B.

[167] Braeuning A., Buchmann A. (2009). The glycogen synthase kinase inhibitor 3-(2,4-dichlorophenyl)-4-(1-methyl-1H-indol-3-yl)-1H-pyrrole-2,5-dione (SB216763) is a partial agonist of the aryl hydrocarbon receptor. Drug Metab. Dispos..

[168] Sekine S., Gutierrez P.J., Lan B.Y., Feng S., Hebrok M. (2007). Liver-specific loss of beta-catenin results in delayed hepatocyte proliferation after partial hepatectomy. Hepatology.

[169] Tan X., Behari J., Cieply B., Michalopoulos G.K., Monga S.P. (2006). Conditional deletion of beta-catenin reveals its role in liver growth and regeneration. Gastroenterology.

[170] Loeppen S., Koehle C., Buchmann A., Schwarz M. (2005). A beta-catenin-dependent pathway regulates expression of cytochrome P450 isoforms in mouse liver tumors. Carcinogenesis.

[171] Gerbal-Chaloin S., Dume A.S., Briolotti P., Klieber S., Raulet E., Duret C., Fabre J.M., Ramos J., Maurel P., Daujat-Chavanieu M. (2014). The WNT/beta-catenin pathway is a transcriptional regulator of CYP2E1, CYP1A2, and aryl hydrocarbon receptor gene expression in primary human hepatocytes. Mol. Pharmacol..

[172] Chesire D.R., Dunn T.A., Ewing C.M., Luo J., Isaacs W.B. (2004). Identification of aryl hydrocarbon receptor as a putative Wnt/beta-catenin pathway target gene in prostate cancer cells. Cancer Res..

[173] Kasai S., Ishigaki T., Takumi R., Kamimura T., Kikuchi H. (2013). Beta-catenin signaling induces CYP1A1 expression by disrupting adherens junctions in Caco-2 human colon carcinoma cells. Biochim. Biophys. Acta.

[174] Vaas S., Kreft L., Schwarz M., Braeuning A. (2014). Cooperation of structurally different aryl hydrocarbon receptor agonists and beta-catenin in the regulation of CYP1A expression. Toxicology.

[175] Braeuning A., Sanna R., Huelsken J., Schwarz M. (2009). Inducibility of drug-metabolizing enzymes by xenobiotics in mice with liver-specific knockout of Ctnnb1. Drug Metab. Dispos..

[176] Ziegler N., Awwad K., Fisslthaler B., Reis M., Devraj K., Corada M., Minardi S.P., Dejana E., Plate K.H., Fleming I. (2016). beta-Catenin Is Required for Endothelial Cyp1b1 Regulation Influencing Metabolic Barrier Function. J. Neurosci..

[177] Zhu X., Gao H., Qin S., Liu D., Cairns J., Gu Y., Yu J., Weinshilboum R.M., Wang L. (2022). Testis- specific Y-encoded- like protein 1 and cholesterol metabolism: Regulation of CYP1B1 expression through Wnt signaling. Front. Pharmacol..

[178] Schulthess P., Loffler A., Vetter S., Kreft L., Schwarz M., Braeuning A., Bluthgen N. (2015). Signal integration by the CYP1A1 promoter—A quantitative study. Nucleic. Acids. Res..

[179] Colletti M., Cicchini C., Conigliaro A., Santangelo L., Alonzi T., Pasquini E., Tripodi M., Amicone L. (2009). Convergence of Wnt signaling on the HNF4alpha-driven transcription in controlling liver zonation. Gastroenterology.

[180] Mulholland D.J., Dedhar S., Coetzee G.A., Nelson C.C. (2005). Interaction of nuclear receptors with the Wnt/beta-catenin/Tcf signaling axis: Wnt you like to know?. Endocr. Rev..

[181] Yang Y., Filipovic D., Bhattacharya S. (2022). A Negative Feedback Loop and Transcription Factor Cooperation Regulate Zonal Gene Induction by 2, 3, 7, 8-Tetrachlorodibenzo-p-Dioxin in the Mouse Liver. Hepatol. Commun..

[182] Caliskan C., Yuce Z., Sercan H.O. (2023). Dvl proteins regulate SMAD1, AHR, mTOR, BRD7 protein expression while differentially regulating canonical and non-canonical Wnt signaling pathways in CML cell lines. Gene.

[183] Kabatkova M., Zapletal O., Tylichova Z., Neca J., Machala M., Milcova A., Topinka J., Kozubik A., Vondracek J. (2015). Inhibition of beta-catenin signalling promotes DNA damage elicited by benzo[a]pyrene in a model of human colon cancer cells via CYP1 deregulation. Mutagenesis.

[184] Amit S., Hatzubai A., Birman Y., Andersen J.S., Ben-Shushan E., Mann M., Ben-Neriah Y., Alkalay I. (2002). Axin-mediated CKI phosphorylation of beta-catenin at Ser 45: A molecular switch for the Wnt pathway. Genes. Dev..

[185] Lee R., Li J., Li J., Wu C.J., Jiang S., Hsu W.H., Chakravarti D., Chen P., LaBella K.A., Li J. (2022). Synthetic Essentiality of Tryptophan 2,3-Dioxygenase 2 in APC-Mutated Colorectal Cancer. Cancer Discov..

[186] Krkoska M., Svobodova J., Kabatkova M., Zapletal O., Vaculova A.H., Nekvindova J., Vondracek J. (2021). Deregulation of signaling pathways controlling cell survival and proliferation in cancer cells alters induction of cytochrome P450 family 1 enzymes. Toxicology.

[187] Qin S., Liu D., Kohli M., Wang L., Vedell P.T., Hillman D.W., Niu N., Yu J., Weinshilboum R.M., Wang L. (2018). TSPYL Family Regulates CYP17A1 and CYP3A4 Expression: Potential Mechanism Contributing to Abiraterone Response in Metastatic Castration-Resistant Prostate Cancer. Clin. Pharmacol. Ther..

[188] Li F., Zhu W., Gonzalez F.J. (2017). Potential role of CYP1B1 in the development and treatment of metabolic diseases. Pharmacol. Ther..

[189] Neumeyer V., Brutau-Abia A., Allgauer M., Pfarr N., Weichert W., Falkeis-Veits C., Kremmer E., Vieth M., Gerhard M., Mejias-Luque R. (2021). Loss of RNF43 Function Contributes to Gastric Carcinogenesis by Impairing DNA Damage Response. Cell Mol. Gastroenterol. Hepatol..

